# LncRNA AK023948 is a positive regulator of AKT

**DOI:** 10.1038/ncomms14422

**Published:** 2017-02-08

**Authors:** Pratirodh Koirala, Jianguo Huang, Tsui-Ting Ho, Fangting Wu, Xianfeng Ding, Yin-Yuan Mo

**Affiliations:** 1 Cancer Institute, University of Mississippi Medical Center, Jackson, Mississippi 39216, USA; 2 Department of Biochemistry, University of Mississippi Medical Center, Jackson, Mississippi 39216, USA; 3 Department of Pharmacology/Toxicology, University of Mississippi Medical Center, Jackson, Mississippi 39216, USA; 4 Department of Radiation Oncology, University of Mississippi Medical Center, Jackson, Mississippi 39216, USA; 5 System Biosciences, Mountain View, California 94041, USA; 6 College of Life Sciences, Zhejiang Sci-Tech University, Hangzhou 310022, China

## Abstract

Despite the overwhelming number of human long non-coding RNAs (lncRNAs) reported so far, little is known about their physiological functions for the majority of them. The present study uses a CRISPR/Cas9-based synergistic activation mediator (SAM) system to identify potential lncRNAs capable of regulating AKT activity. Among lncRNAs identified from this screen, we demonstrate that AK023948 is a positive regulator for AKT. Knockout of AK023948 suppresses, whereas rescue with AK023948 restores the AKT activity. Mechanistically, AK023948 functionally interacts with DHX9 and p85. Importantly, AK023948 is required for the interaction between DHX9 and p85 to hence the p85 stability and promote AKT activity. Finally, AK023948 is upregulated in breast cancer; interrogation of TCGA data set indicates that upregulation of DHX9 in breast cancer is associated with poor survival. Together, this study demonstrates two previously uncharacterized factors AK023948 and DHX9 as important players in the AKT pathway, and that their upregulation may contribute to breast tumour progression.

## Abstract (web summary)

The function of many human long non-coding RNAs (lncRNAs) is still undetermined. Here, the authors setup a gain of function CRISPR-based screen and identify a lncRNA that positively regulates AKT activity by interacting with the RNA helicase DHX9 resulting in stabilization of PI3K regulatory subunit p85.

Advances in functional genomics have revealed that the human genome is actively transcribed; however, vast majority of the transcripts are non-coding RNA including microRNAs and long non-coding RNAs (lncRNAs)^1^. Unlike microRNAs, lncRNAs are larger than 200 bp in length, and some of them may be capped and polyadenylated. Increasing evidence suggests that lncRNAs could be the key regulators of different cellular processes. Various mechanisms have been proposed to explain how lncRNAs may have an impact on gene expression. One of well-characterized mechanisms is the lncRNA-mediated gene regulation through interaction with DNA, RNA or protein. For instance, HOTAIR acts as a scaffold to recruit proteins required for chromatin remodelling^2^. On the other hand, GAS5 imitates glucocorticoid response element and binds to glucocorticoid receptor such that it prevents from binding to its response element^3^. In addition, GAS5 inhibits the expression of miR-21 through the competing endogenous RNA mechanism^4^. There are many other examples of lncRNAs as scaffolds that bring together multiple proteins to form functional ribonucleoprotein complexes^5^,^6^,^7^,^8^. Through interactions with different binding partners, lncRNAs can regulate their function, stability or activity.

The phosphoinositide-3-kinase (PI3K)–protein kinase B/AKT (PI3K-PKB/AKT) pathway is at the centre of cell signalling; it responds to growth factors, cytokines and other cellular stimuli. Once activated, AKT transfers signaling and regulates an array of downstream targets including well-known MDM2/p53, Foxo and NF-κB. As a result, AKT plays a key role in the diverse cellular processes, including cell survival, growth, proliferation, angiogenesis, metabolism and cell migration^9^. The AKT activity can be influenced by many factors, such as growth factors or their corresponding receptors, causing different biological consequences^10^. Among them, PI3K and PTEN are major regulators of AKT^11^,^12^. Evidence indicates that AKT is often dysregulated in cancer^13^; however, the underlying mechanism is still not fully understood despite many years of investigations. In particular, it is not known whether lncRNAs are involved in the regulation of AKT activity.

Given the critical role of AKT in cell signalling, we design a screen system based on CRISPR/Cas9 synergistic activation mediator (SAM)^14^ and an AKT reporter to identify lncRNAs as AKT regulators. Through this screen, validation and further characterization we show that AK023948 positively regulates AKT activity by interaction with DHX9 and the regulatory subunit of PI3K.

## Results

### AK023948 as a positive AKT regulator

A variety of utilities of CRISPR/Cas9 system have been explored such as gene activation^15^ or repression^16^. Regarding gene activation, a recently reported SAM system uses MS2 bacteriophage coat proteins combined with p65 and HSF1, and it significantly enhances the transcription activation^14^. Therefore, we adopted this system for lncRNAs and designed gRNAs (five gRNAs for each lncRNA) covering ∼1 kb upstream of the first exon to activate the endogenous lncRNAs. We focused on a specific group of lncRNAs (Supplementary Data set 1) primarily based on two sources ( www.lncrandb.org and http://www.cuilab.cn/lncrnadisease).

**Figure 1:**
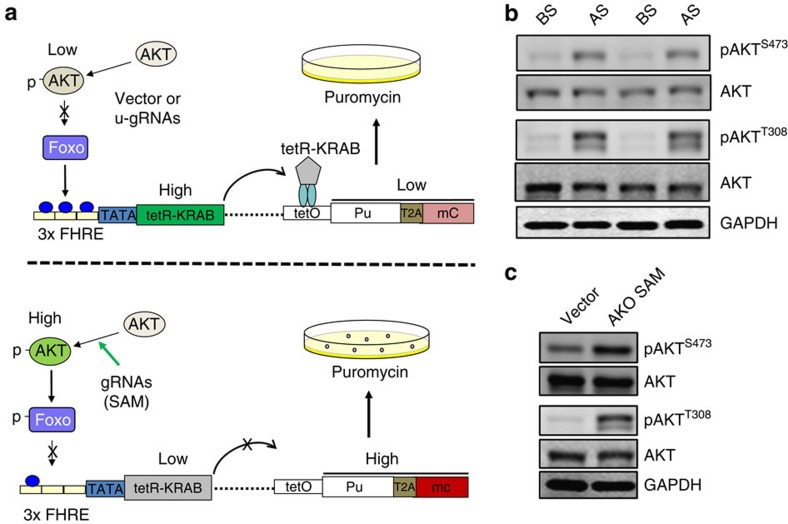
Identification of lncRNAs capable of activating AKT by SAM library screen along with an AKT reporter. (**a**) Schematic description of principle for the AKT reporter used to screen the SAM library. SAM gRNAs that can activate AKT activity are enriched by puromycin selection. (**b**) Detection of AKT activity for cells before selection (BS) and cells after selection (AS) by western blot. (**c**) AK023948 SAM gRNA increases the pAKT level. A mixed pool of five SAM gRNAs against AK023948 were introduced into MCF-7 cells carrying dCas9-VP64 and pMS2-p65-HSF1 by infection and cellular extract was prepared for western blot 2 days after infection.

For screening, we designed an AKT reporter (Fig. 1a) because the AKT pathway is at the centre of cell signaling. This reporter system takes advantage of the Foxo transcription factors as direct targets of AKT and is capable of binding to forkhead response elements. Phosphorylation of Foxo by pAKT causes subcellular redistribution of Foxo, followed by rapid degradation^17^. Thus, the reporter vector carries three copies of forkhead response element at the upstream of the well-known fusion repressor tetR-KRAB, which binds to the corresponding tet operator (tetO)^18^,^19^,^20^ in the same vector. The tetO controls the puromycin gene (Pu) and mCherry (tetO-Pu-T2A-mC). It is able to confer resistance to puromycin when no tetR-KRAB is bound on the tetO site. However, when tetR-KRAB binds to the tetO site, Pu is suppressed and the cells carrying this reporter become sensitive to puromycin. Since vector control or unrelated gRNAs (u-gRNAs) have no effect on pAKT and the level of Pu is low because of suppression by tetR-KRAB, few cells are expected to survive (Fig.1a, top). However, if a certain gRNA can induce lncRNAs, which are capable of activating AKT (Fig. 1a, bottom), these cells are expected to survive and proliferate because little tetR-KRAB binds to the tetO site, and they are resistant to puromycin.

A screen procedure was outlined in Supplementary Fig. 1. After selection against puromycin, surviving cells were harvested. Compared with the cells before selection, after selection cells displayed an increase in pAKT at both T308 and S473 (Fig. 1b). Total RNA was extracted from the pooled after selection cells and before selection cells; lncRNA profiling by reverse transcriptase PCR (RT–PCR) arrays revealed that several candidate lncRNAs (Supplementary Fig. 2A) including AK023948 were upregulated presumably because of function of the corresponding SAM gRNAs. We chose AK023948 (the top of list) for further characterization. We confirmed that AK023948 gRNAs were able to increase the endogenous AK023948 (Supplementary Fig. 2B). In addition, AK023948 gRNAs conferred puromycin resistance for the cells carrying the AKT reporter (Supplementary Fig. 2C) and enhanced AKT activity (Fig. 1c).

### AK023948-mediated AKT activation

**Figure 2:**
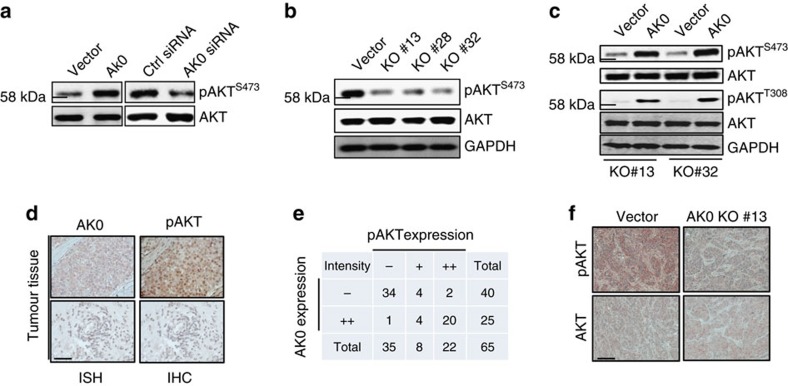
AK023948 regulates the pAKT level. (**a**) While AK023948 overexpression increases, AK023948 knockdown decreases phosphorylation of AKT. MCF-7 cells were transfected with either AK023948 expression vector or AK023948 siRNA. Cellular extract was prepared for western blot 48 h after transfection. (**b**) AK023948 KO suppresses the pAKT level. AK023948 KO was performed in MCF-7 using a procedure as described in Methods. (**c**) Re-expression of AK023948 in the AK023948 KO cells increases the pAKT level. AK023948 expression vector or control vector was introduced into KO cells, and total cellular extract was prepared for western blot. (**d**) High levels of AK023948 and pAKT in breast tumour specimens with representative images for the same field of a tumour. The same TMA was first detected for AK023948 by ISH and then the signal was stripped, followed by IHC to detect pAKT. Scale bar, 100 μm. (**e**) A positive correlation between AK023948 and pAKT from the same TMA with *P*-value of Fisher's exact test for the association <0.0001. (**f**) The pAKT level is lower in xenograft tumours derived from AK023948 KO than in vector control tumours, as detected by IHC. Scale bar, 50 μm.

AK023948 is a single exon gene with 2,807 bp (ref. 21). Further experiments with manipulation of AK023948 expression demonstrated that AK0023948 promoted the AKT activity. For example, ectopic expression of AK023948 (Supplementary Fig. 3A) increased the pAKT level (Fig. 2a, left). In contrast, suppression of AK023948 by RNA interference (RNAi; Supplementary Fig. 3B) reduced the pAKT level (Fig. 2a, right; Supplementary Fig. 3C). To better determine the role of AK023948 in AKT activation, we generated AK023948 knockout (KO) by CRISPR/Cas9 using a dual gRNA approach^22^ (Supplementary Fig. 4). This AK023948 effect on AKT was more obvious in AK023948 KO clones (Fig. 2b). To further determine the role of AK023948 in activation of AKT, we re-expressed AK023948 in the KO cells, that is, rescue experiment. In both KO clones, re-expression of AK023948 was able to enhance the pAKT level (Fig. 2c; Supplementary Fig. 5A). In consistence with this finding, AK023948 KO also caused downregulation of the phosphorylation of PDK1 (Supplementary Fig. 5B), a known immediate upstream kinase of AKT, and re-expression of AK023948 was able to enhance PKD1 phosphorylation (Supplementary Fig. 5B). Although AK023948 is imbedded in thyroglobulin (TG) and Src-like-adaptor (SLA)^21^, we found little effect of AK023948 KO on SLA, as determined by qRT–PCR (Supplementary Fig. 5C), and TG was undetectable in these cells. In addition, we detected a positive correlation between AK023948 and pAKT in breast cancer tissue microarrays (TMA), where we first detected AK023948 by *in situ* hybridization (ISH; Fig. 2d) and then treated the previously ISH-stained TMAs by the acid/alcohol method^23^,^24^ to remove the AK023948 signal (Supplementary Fig. 6) before immunohistochemistry (IHC) staining for pAKT (Fig. 2d). For instance, 34 of 65 cases revealed low levels of both AK023948 and pAKT and 20 of 65 cases were high for both AK023948 and pAKT (Fig. 2e). Finally, IHC analysis of xenograft tumours derived from AK023948 KO or vector control indicated that AK023948 KO caused a remarkable reduction of the pAKT level (Fig. 2f).

### Role of DHX9 in the AK023948-mediated AKT activation

**Figure 3:**
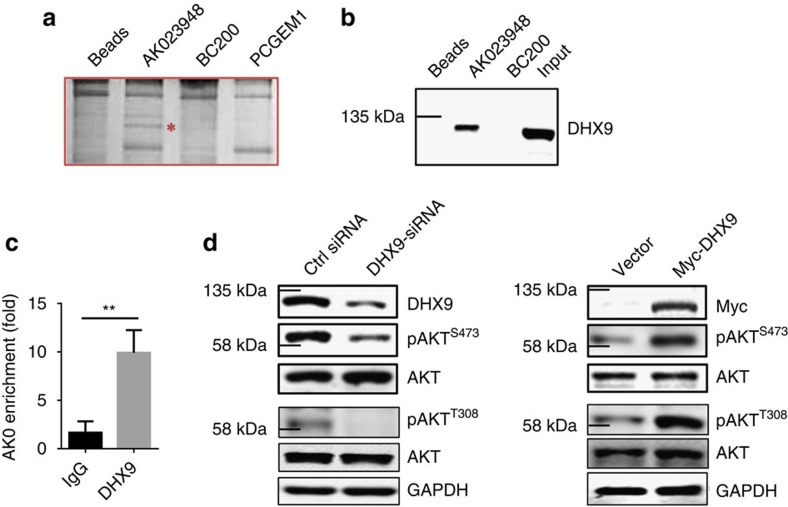
DHX9 is an AK023948-binding partner and is involved in regulation of AKT activity. (**a**) RNA precipitation using the biotin-labelled AK023948 probe, followed by PAGE and silver staining. Red star indicates a unique band bound to AK023948. Mass spectrometry analysis suggested DHX9 as a candidate. (**b**) Confirmation of the interaction between AK023948 and DHX9 by RNA precipitation and western blot. (**c**) Confirmation of the interaction between AK023948 and DHX9 by RNA immunoprecipitation using DHX9 antibody. (**d**) While DHX9 siRNA suppresses, ectopic expression of DHX9 increases the pAKT level. DHX9 siRNAs or DHX9 expression vector was introduced into MCF-7 cells by transfection, and cellular extract was prepared for western blot 48 h after transfection. Values in **c** are s.e.m. (*n*=3). **P<0.01 by two-tailed Student's *t*-test.

To determine the underlying mechanism of AK023948-mediated AKT activation, we performed RNA precipitation using a biotin-labelled AK023948 RNA probe. When the precipitate was subject to SDS–PAGE analysis, followed by silver staining, we detected a unique band of ∼140 kDa to AK023948, which was missing in precipitates from either BC200 or PCGEM1 as a control probe (Fig. 3a; Supplementary Fig. 7). Mass spectrometry analysis of this unique band was suggested as ATP-dependent RNA helicase A (RHA/DHX9). This result was confirmed by western blot analysis of the same RNA precipitate (Fig. 3b). To further confirm their interaction, we performed RNA immunoprecipitation (RIP) using DHX9 antibody and detected over a 10-fold enrichment of AK023948 with DHX9 antibody over IgG control (Fig. 3c). To determine the role of DHX9 in AKT activation, we suppressed DHX9 by RNAi and ectopically expressed DHX9, respectively. We showed that DHX9 siRNAs reduced pAKT, whereas ectopic expression of DHX9 increased pAKT (Fig. 3d), suggesting that DHX9 is involved in the AKT pathway and interaction of DHX9 with AK023948 may be required for the AK023948-mediated AKT activation.

### AK023948 is required for the interaction of DHX9 with p85

**Figure 4:**
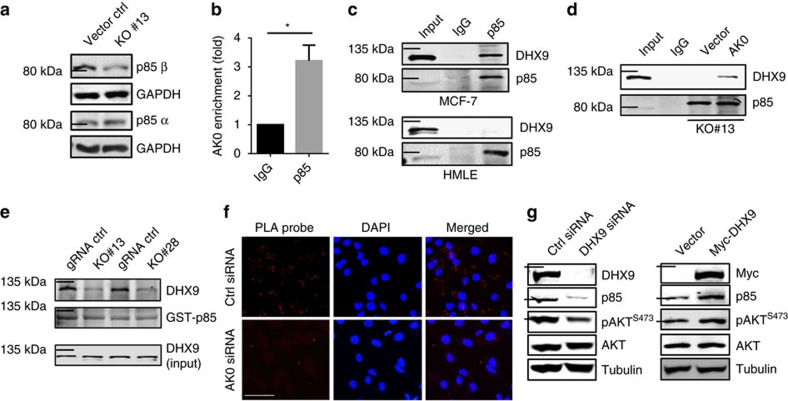
AK023948 is required for the interaction between DHX9 and p85. (**a**) AK023948 KO primarily suppresses the p85β level, as detected by western blot. (**b**) AK023948 interacts with p85, as detected by RIP assay using p85 antibody. (**c**) AK023948 is required for the interaction between DHX9 and p85, as detected by co-immunoprecipitation (co-IP) using p85 antibody. HMLE cells express little AK023948, whereas MCF-7 cells express a high level of AK023948. (**d**) Rescue experiments further suggest that AK023948 is critical for the interaction between AK023948 and p85. (**e**) AK023948 is required for the interaction between DHX9 and p85, as determined by GST pulldown assay. The DHX9 level pulled down by GST-p85 was lower in KO cells than in gRNA control. (**f**) AK023948 is required for the interaction between DHX9 and p85, as detected by the Duolink *in situ* Fluorescence Kit (Sigma). HeLa cells were transfected with Myc-p85 plus control siRNA or AK0 siRNA. After 48 h, the cells were fixed for PLA. The red signals were lower in AK0 siRNA than in control siRNA cells. Scale bar, 100 μm. (**g**) DHX9 siRNAs suppress, but ectopic expression of DHX9 increases both p85 and pAKT. Values in **b** are s.e.m. (*n*=3). *P<0.05 by two-tailed Student's *t*-test.

DHX9 is a RNA helicase carrying various domains such as RBD1, RBD2, RGGG and helicase^25^. In addition, DHX9 interacts with double-stranded nucleic acids and protein factors like NF-κB p65 (ref. 26) and BRCA1 (ref. 27). In particular, DHX9 might also bind to the regulatory subunit of PI3K, p85, as suggested by mass spectrometry analysis^28^. It is well known that PI3K consists of a regulatory subunit p85 and a catalytic subunit p110, and together they regulate AKT activity^29^. With regard to the regulatory subunit, there are two major isoforms of p85, that is, p85α and p85β (ref. 13), and thus, we determined their relative expression levels. As shown in Supplementary Fig. 8A, we detected a fair amount of p85β with p85β antibody, but detected little p85α with p85α antibody in MCF-7 cells. Of interest, AK023948 KO suppressed the p85β level, but had little effect on p85α (Fig. 4a; Supplementary Fig. 9A), suggesting that p85β is a major target of AK023948. Western blot using pan-p85 antibody also only detected p85β isoform in 293T and MCF-7 cells (Supplementary Fig. 8B). Therefore, we performed RIP assays using the same pan-p85 antibody in the following experiments. As expected, AK023948 interacted with p85, revealing over a 3-fold enrichment as compared with IgG control (Fig. 4b). In addition, RNA precipitation with AK023948 as a probe also detected p85 AK023948, and both DHX9 and p85 can bind to the 3′ region of AK023948 (Supplementary Fig. 9B).

Co-immunoprecipitation assays confirmed the interaction of DHX9 with p85 in MCF-7 cells (Fig. 4c, top). Importantly, AK023948 is required for this interaction. For example, HMLE cells express little AK023948 (see Fig. 6b); no visible interaction between DHX9 and p85 was detected (Fig. 4c, bottom). Furthermore, AK023948 KO abolished this interaction in MCF-7 cells (Fig. 4d, vector lane). Re-expression of AK023948 in the AK023948 KO cells was able to restore the ability of p85 to interact with DHX9 (Fig. 4d, last lane on the right). Glutathione S-transferase (GST) pulldown assays further indicated that AK023948 is required for the interaction between DHX9 and p85 because the amount of DHX9 pulled down by GST-p85 was lower in AK023948 KO than in gRNA control (Fig. 4e). Moreover, proximity ligation assay (PLA) revealed that suppression of AK023948 by RNAi reduced the interaction between DHX9 and p85 (Fig. 4f; Supplementary Fig. 9C). Finally, DHX9 siRNAs suppressed both p85 and pAKT, whereas ectopic expression of DHX9 enhanced the p85 and pAKT level (Fig. 4g).

### AK023948 promotes the p85 stability

**Figure 5:**
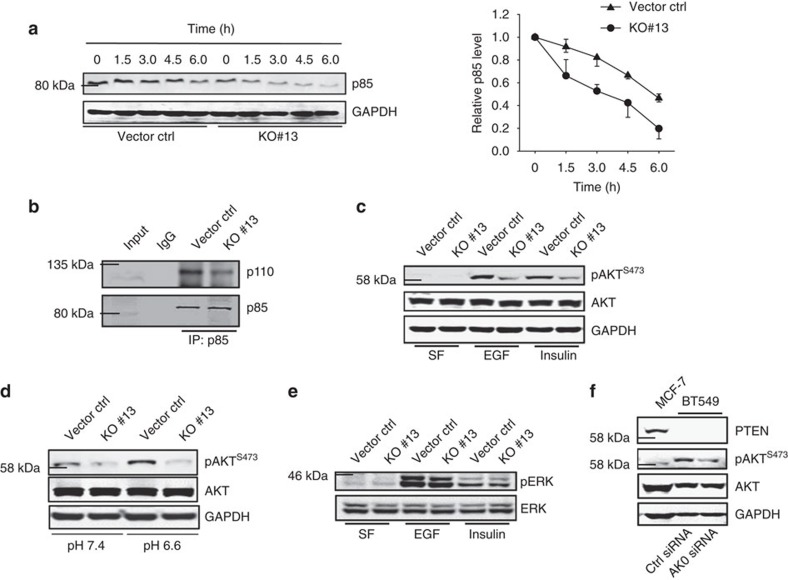
AK023948 has an impact on the p85 stability. (**a**) Effect of AK023948 KO on the p85 stability. MCF-7 cells were treated with cycloheximide (CHX) at 20 μg ml^−1^ and then were harvested for western blot at indicated time points. Half-life curve is on the right. (**b**) AK023948 KO reduces the interaction of p85 with p110, as detected by co-IP. (**c**) AK023948 KO inhibits the growth factor-induced AKT activation. Cells were first cultured in a serum-free medium for 2 h and then EGF or insulin was added at 10 ng ml^−1^ for 30 min. (**d**) AK023948 KO inhibits the acidosis-induced AKT activation. Cells were cultured at pH 7.4 or pH 6.6 for 2 h before harvesting for western blot. (**e**) AK023948 KO has no effect on ERK activity. Cells were treated with the same way as in **c**. (**f**) Suppression of AK023948 by RNAi can still reduce the pAKT level in PTEN-deficient BT549 cells.

We further showed that AK023948 and DHX9 regulated the stability of p85. For example, the half-life of p85 was ∼5.5 h for vector control, whereas it was ∼3.5 h in AK023948 KO cells (Fig. 5a). Similarly, DHX9 siRNAs also decreased the p85 stability (Supplementary Fig. 10A) and p85 short interfering RNA (siRNA) reduced AKT activity (Supplementary Fig. 10B). However, DHX9 had no effect on AK02398 expression (Supplementary Fig. 11A) or its subcellular distribution (Supplementary Fig. 11B). Together, these results suggest that both AK023948 and DHX9 are involved in the regulation of the p85 stability.

Next, we showed that AK023948 affected the interaction between p85 and p110 because AK023948 KO reduced their interaction (Fig. 5b). Receptor tyrosine kinases (RTKs) are major kinases responsible for activation of PI3K, which then converts phosphatidylinositol-3,4-diphosphate to phosphatidylinositol (3,4,5)-trisphosphate to serve as a secondary messenger. To determine the role of AK023948 in RTK-mediated AKT activation, we first cultured the cells in serum-free medium for 2 h and then treated the cells with epidermal growth factor (EGF) or insulin at 10 ng ml^−1^. As expected, no pAKT was detected during serum starvation, and EGF or insulin induced AKT activity (Fig. 5c). However, this EGF/insulin-induced AKT activation was severely inhibited in AK023948 KO cells (Fig. 5c). In addition, we starved the cells for 12 h and then added insulin for 5∼90 min. Again, the insulin-induced AKT activity was substantially decreased in AK023948 KO cells as compared with gRNA control (Supplementary Fig. 12A,B). Similarly, acidosis-induced AKT activity^30^ was also impaired in AK023948 KO cells (Fig. 5d; Supplementary Fig. 12C).

ERK is a downstream target of RAS and RTKs. After activation, RTKs can also recruit RAS which subsequently interacts with the catalytic subunit of PI3K, p110, to activate AKT^31^,^32^,^33^. Of interest, AK023948 KO had little effect on the pERK level whether EGF/insulin is absent or present (Fig. 5e; Supplementary Fig. 12A,B), providing additional evidence that AK023948 acts on p85, but not p110, to activate AKT. PTEN is a well-known tumour suppressor^34^. As a phosphatase, PTEN reverses PI3P to PI2P and thus functions as a suppressor for AKT. BT549 is a PTEN-deficient cell line such that the pAKT level is high as compared with that in MCF-7 cells (Fig. 5f). However, AK023948 siRNA was still able to suppress the pAKT level (Fig. 5f; Supplementary Fig. 12D). Finally, AKT inhibitors such as PP2A^35^ can act on AKT; suppression of PP2A by okadaic acid increased pAKT levels^36^; however, the induction was lower in KO cells than in control cells (Supplementary Fig. 12E).

### Clinical significance of AK023948 and DHX9

**Figure 6:**
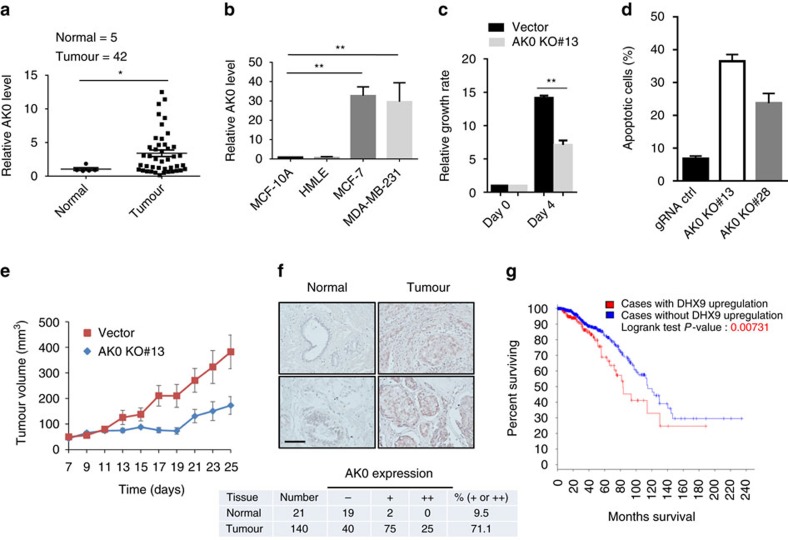
Upregulation of AK023948 in breast cancer and promotion of tumorigenesis. (**a**) Detection of AK023948 in the OriGene breast cancer tissue cDNA array by qPCR. (**b**) AK023948 is upregulated in breast cancer cells (MCF-7 and MDA-MB-231) as compared with non-malignant breast cells (MCF-10A and HMLE). (**c**) AK023948 KO suppresses cell proliferation of MCF-7, as detected by MTT assay. (**d**) AK023948 KO promotes apoptosis. Cells were seeded in slide chambers, and treated with H_2_O_2_ at 0.8 mM for 4 h before TUNEL assay. Apoptotic cells were counted from three different fields, and apoptotic cell ratio was calculated against total cells. (**e**) Tumour growth for vector control and AK0 KO#13 in nude mice. (**f**) Expression of AK023948 in breast cancer TMAs, as detected by ISH. Scale bar, 100 μm. Bottom: quantitative analysis of AK023948 expression based on the results from **f**. (**g**) Upregulation of DHX9 expression is associated with poor patient survival. About 20.9% (229 of 1091 cases analysed) are positive for DHX9 using the Onco Query Language (OQL; EXP>1.5). Values in **b**,**c** are s.e.m. (*n*=3). **P<0.01 by two-tailed Student's *t*-test.

In consistence with upregulation of AKT activity by AK023948, we showed that AK023948 was highly expressed in breast tumour tissue compared with normal breast tissue (Fig. 6a). Moreover, the AK023948 level was also higher in cancer cell lines MCF-7 and MDA-MB-231 cells than in non-malignant cells HMLE and MCF-10A (Fig. 6b). These results suggest that AK023948 may play an oncogenic role. Indeed, MTT assays indicated that AK023948 siRNA significantly suppressed cell growth *in vitro* (Supplementary Fig. 13A). In contrast, ectopic expression of AK023948 promoted cell growth (Supplementary Fig. 13B). Furthermore, cell growth for AK023948 KO cells was significantly reduced compared with gRNA control (Fig. 6c; Supplementary Fig. 13C) and AK023948 KO caused more apoptosis (Fig. 6d; Supplementary Fig. 14). Experiments with xenograft mouse model revealed that AK023948 KO remarkably inhibited tumour growth rate (Fig. 6e) and tumour weight (Supplementary Fig. 15A). In consistence with this result, IHC analysis of xenograft tumours revealed that AK023948 KO caused a substantial decrease in the level of the proliferation marker Ki-67 (Supplementary Fig. 15B). ISH of breast TMA provided further evidence that AK023948 was upregulated in breast tumours as compared to normal tissue (Fig. 6f). For example, 71% of breast tumour specimens was positive for AK023948, whereas only 9.5% of normal breast tissue was positive for AK023948.

Given the role of DHX9 in AKT activity, we interrogated the Cancer Genome Atlas-invasive breast carcinoma data set at cBioportal ( http://www.cbioportal.org/)^37^,^38^ and found that DHX9 was upregulated in 21% of 1,091 cases (Supplementary Fig. 16A, top). Importantly, this upregulation of DHX9 was positively associated with poor overall survival (Fig. 6g). For example, median months survival for cases with DHX9 upregulation were 83.25, whereas the median months survival for cases without DHX9 upregulation were 114.73 (Supplementary Fig. 16A, bottom). Furthermore, DHX9 was also upregulated in breast cancer cell lines as compared with non-malignant HMLE cells (Supplementary Fig. 16B). Together, these results highlight the clinical significance of AK023948 and DHX9.

## Discussion

Activation of the AKT pathway in cancer has been extensively investigated in past decades^13^, which has demonstrated a critical role of PI3K in tumorigenesis by regulation of AKT activity. While it is well known now that oncogenic mutations in PI3K genes can contribute to upregulation of AKT activity in cancer, little is known how PI3K itself is regulated. In the present study, we provide evidence that AK023948 is required for AKT activation through interaction with DHX9 and p85. Our study suggests that the interaction of AK023948 with p85 and DHX9 is critical for AKT activation in response to growth factors and environmental stress such as acidosis. Therefore, AK023948 and DHX9 are previously uncharacterized important players in the AKT pathway.

DHX9 is a DEAD box protein with RNA helicase activity^25^. It may participate in melting of DNA:RNA hybrids, such as those that occur during transcription. Of interest, DHX9 can interact with many proteins including BRCA1 (ref. 27), KHDRBS1 (ref. 39), AKAP8L (ref. 40) and NXF1 (ref. 41). Thus, DHX9 is implicated in a number of cellular processes involving alterations of RNA secondary structure such as translation initiation, nuclear and mitochondrial splicing, and ribosome and spliceosome assembly. However, up to date there is no evidence that DHX9 is involved in AKT activity. Our study demonstrates that as an AK023948-binding partner, DHX9 along with AK023948, regulates the p85 stability. For example, AK023948 KO or suppression of DHX9 by RNAi significantly decreases the half-life of p85.

Activation of AKT is a complicated process, which involves vast arrays of players, starting at RTK. Upon activation of RTK by growth factors, PI3K is capable of generating the second messenger phosphatidylinositol (3,4,5)-trisphosphate, which subsequently activates critical downstream targets such as AKT and mammalian target of rapamycin. When dysregulated, the PI3K pathway has a causal role in many forms of cancer, including breast cancer^42^. Despite the importance of p85 in the AKT pathway, information about the regulation of p85 is limited. Nevertheless, a recent report suggests that the short isoform of ErbB3-binding protein 1, p42, is able to increase degradation of p85, leading to a decreased AKT activity and tumorigenesis^43^. Our study suggests an additional mechanism by which AK023948 and DHX9 can regulate the stability of p85. Given that DHX9 interacts with AK023948, it is conceivable that activation of the PI3K complex requires both AK023948 and DHX9.

The PI3K regulatory subunit consists of two major isoforms, that is, p85α and p85β, but evidence indicates that they have an opposite effect on AKT activity and tumorigenesis. For instance, KO experiments suggest that p85α functions as a tumour suppressor^44^, which is also supported by the findings that p85α is downregulated in cancer specimens^45^,^46^. Moreover, p85α deletion increases tumorigenesis. A functional missense mutation in p85α resulting in reduced p85 expression is associated with colon cancer^47^. In contrast to p85α, p85β plays an oncogenic role. Upregulation of p85β is found in several cancers, and in an experimental setting p85β drives tumour progression^46^. Expression of p85β induces oncogenic transformation of primary avian fibroblasts^48^. In support of these findings, we show that p85β is a primary target for AK023948 in breast cancer cells.

Although lncRNAs are poorly characterized in general, emerging evidence indicates that lncRNAs may function as oncogenes and tumour suppressors, thus having an impact on one or more of the cancer hallmarks^49^. While much has been learned in the past years about the role of a small number of lncRNAs in cancer such as HOTAIR, little is known about AK023948. In this regard, our study suggests an oncogenic role of AK023948 in breast cancer, in contrast to what has been reported in papillary thyroid cancer^21^. This is based on these lines of evidence. (1) AK023948 is upregulated in breast cancer and its expression is correlated with pAKT in clinical specimens. (2) While ectopic expression of AK023948 promotes, AK023948 siRNA or AK023948 KO suppresses AKT activity, and tumour cell growth. (3) Re-expression of AK023948 in AK023948 KO cells is able to restore the AKT activation. (4) As an AK023948-binding partner, DHX9 is also upregulated in breast tumours; this upregulation is associated with poor overall survival. Importantly, our results further suggest that this AK023948-mediated AKT activation is at least in part through interaction with DHX9 and p85. Hence, these results provide a molecular basis that AK023948 plays an oncogenic role in breast cancer.

In summary, our screen system provides a platform for identification of AKT-associated lncRNAs. Furthermore, our study suggests that AK023948 functions as a positive regulator for AKT (Supplementary Fig. 17). AK023948 is required for the interaction DHX9 and p85, as suggested co-immunoprecipitation and GST pulldown assays, and is also critical to AKT activity in response to various stimuli such as growth factors or acidosis. In normal cells, the AK023948 level is low, and thus, the AKT activity is low even in the presence of growth factors. This low AKT activity may also be attributed to the low level of DHX9. In the tumour cells, upregulation of AK023948 and DHX9 leads to a high activity of AKT. Several reports indicate that upregulation or mutation in p85 and p110 may contribute to an elevated level of pAKT and cancer development^50^,^51^. Our results suggest an additional mechanism for regulation of AKT activity. Thus, these tumour cells become more proliferative and aggressive.

## Methods

### Reagents

Primary antibodies were purchased from the following sources: pAKT^T473^ (#4060), pAKT^S308^ (#2965), AKT (#2920), p85 (#4257), p110 (#4249), pERK (#4370), ERK (#4696) from Cell Signaling (Danvers, MA); DHX9 (#26271), p85α (#191606) and p85β (#180917) from Abcam (Cambridge, MA). Most of western blots were performed at 1,000 × dilution of primary antibodies. GAPDH and tubulin from ProteinTech (Chicago, IL), PTEN (#7974) was from Santa Cruz (Dallas, TX). Secondary antibodies conjugated with IRDye 800 CW or IRDye 680 were purchased from LI-COR Biosciences (Lincoln, NE). PCR primers were obtained from IDT (Coralville, IA). AK023948 siRNAs and control siRNA were purchased from Fisher Scientific (Pittsburgh, PA). DHX9 siRNAs (#sc-45706, pooled) and p85β siRNAs (#sc-39125, pooled) were purchased from Santa Cruz. Transfection of siRNAs was carried out at 50∼100 nM concentration. AK023948 LNA probe and control oligos for ISH was purchased from Exiqon (2950 Vedbaek, Denmark). Breast cancer TMAs were purchased from US Biomax (Rockville, MD). Commercial cDNA arrays of breast cancer tissues were purchased from OriGene (Rockville, MD).

### Cell culture

Breast cancer cell lines MCF-7 and MDA-MB-231 (ATCC, Manassas, VA) were grown in RPMI 1640 (Fisher Scientific) with 10% FBS (Sigma-Aldrich) and 2 mM glutamine. MCF-10 A (ATCC) and HMLE cells (Dr Robert A. Weinberg, The Massachusetts Institute of Technology) were grown in DME/F-12 (Fisher Scientific) with 5% FBS, 2 mM glutamine, along with 20 ng ml^−1^ EGF, 10 μg ml^−1^ insulin and 0.5 μg ml^−1^ hydrocortisone. All the media were supplemented with 100 units of penicillin per ml and 100 μg of streptomycin per ml (Fisher Scientific). MCF-7 cells were used in SAM library screen. MCF-7 and MDA-MB-231 cells were authenticated by DDC Medical ( http://www.ddcmedical.com) using the short tandem repeat profiling method. Mycoplasma test was performed by PCR amplification method (Applied Biological Materials, Richmond, BC, Canada).

### MTT assay

MTT assay was performed to determine the effect of AK023948 on cell growth. Approximately 2,000 cells were seeded in triplicate into 96-well plates and grown for 3 days before MTT assay.

### Transfection

Cells were transfected with plasmid DNA using DNAfectin (Applied Biological Materials) or with siRNAs using RNAfectin reagent (Applied Biological Materials) following the manufacturer's protocol.

### Lentivirus infection

Lentivirus was packaged in 293T cells using pPACK packaging mixture (SBI) and the virus containing culture medium was collected and spun for 10 min at 3,000 r.p.m. for 48 h after transfection. Lentivirus infection was carried out six-well plates by mixing 500 μl virus supernatant+500 μl medium containing 8 μg polybrene at multiplicity of infection<1.

### Western blot

The cells were treated as per requirement, and total protein was harvested form cells using lysis buffer (20 mM Tris, pH 8.0; 100 mM NaCl; 1 mM EDTA; 0.5% NP-40; 10% glycerol). Concentration of protein was estimated by Bradford method^52^. The protein samples were run in SDS–PAGE and probed with an antibody of interest.

### Verification of AK023948 transcript

We performed 5′ RACE using a previous described procedure^53^, followed by PCR with primers AKO-polyT adaptor, AK0-adaptor and AK0-5Race-3.2 (Supplementary Data set 2). For 3′ RACE we first added poly adenosine by poly A polymerase (NEB) and then reverse transcribed using AKO-polyT adaptor primer. PCR used primers AK0-3Race-5.1 and AKO-adaptor-5.1 (Supplementary Data set 2). The resulting PCR product was cloned into pCR8 for DNA sequencing.

### GST pulldown assay

PIK3R2 (p85β) was cloned into pGEX-2T at EcoR1 and BamH1, and GST-p85β fusion protein was purified by Glutathione Sepharose 4B (GE Healthcare Life Sciences) according to the manufacturer's protocol. Cellular extracts from gRNA control or AK0 KO#13 and KO#28 were used for pulldown assays.

### Generation of SAM library

Five gRNAs were designed for each lncRNAs as in shown in Supplementary Data set 1. In addition, 10 gRNAs against non-human genes were designed as negative controls with a total 1,215 gRNAs in this pool. The lncRNA-specific gRNAs were designed using Chop-Chop programme^54^ against ∼1 kb upstream of the first exon of lncRNAs. Oligonucleotides carrying each gRNA were synthesized as a mixed pool (CustomArray Inc.) and then amplified by PCR using primers SAM gRNA-5.1 and SAM gRNA-3.1 and finally cloned into a modified pMS2 vector by Gibson assembly method^55^.

### Screen procedure

We subsequently introduced dCas9-VP64 and pMS2-p65-HSF1 (ref. 14) into MCF-7 cells by infection and established stable transductants. Next, we introduced SAM library or vector control by infection. Finally, we introduced the AKT reporter. Potential clones were selected in the presence of puromycin at 0.5 μg ml^−1^ for 5 days. To determine which gRNAs are enriched, we pooled surviving cells and isolated total RNAs, and then profiled lncRNAs by qRT–PCR using primers against the targeted lncRNAs.

### LncRNA profiling

LncRNA profiling (RT–PCR arrays) was performed in 96-well plates using SYBR Green method. Total RNA was isolated using Direct-zol RNA MiniPrep Kit (Zymo Research, Irvine, CA) as suggested by manufacturer. Reverse transcription was carried out by using RevertAid Reverse Transcriptase (Fisher Scientific) and random primer mix (New England BioLabs, Ipswich, MA). Analysis of qRT–PCR was performed as described previously^56^. Delta–delta Ct values were used to determine their relative expression as fold changes.

### Plasmid construction

Individual AK023948 SAM gRNAs were constructed by first annealing each pair of oligos (Supplementary Data set 2) and ligating them to BsmB1-linearized pSM2 vector^14^. AK023948 expressing vector was generated in pCDH-MSCV-EF1-GFP-T2A-Pu (System Biosciences) using PCR primers AK023948-R1-5.1 and AK023948-Not1-3.1. The same approach was used to clone DHX9 with N-terminal Myc tag. Dual gRNA cloning for AK023948 KO was performed as previously described^22^. In brief, AK023948 donor vector was constructed using primers AK023948-right-R1-5.1 and AK023948-right-R1-3.1; AK023948-left-BamH1-5.1 and AK023948-left-BamH1-3.1. GST-p85 was constructed by PCR using primers pGEX-2 T-BamH1-PI3KR2-5.1 and pGEX-2 T-R1-PI3KR2-3.1, and then cloned into pGEX-2 T at BamHI and EcoRI sites. Myc-p85 was constructed by PCR using primers PI3KR2-R1-Myc-5.1 and PI3KR2-Not1-3.1 and then cloned into pCDH-CMV-EF1-Pu (System Biosciences) at EcoRI and NotI sites. The high-fidelity DNA polymerase Phusion enzyme (New England BioLabs) was used for PCR. All PCR products were verified by DNA sequencing.

### AK023948 KO by CRISPR/Cas9

Selection of AK023948 KO clones in MCF-7 cells was carried out using the procedure as described previously^22^. Briefly, AK023948 dual gRNA and donor vector were co-transfected into the MCF-7 cells in six-well plate using DNAfectin (Applied Biological Materials). Next day the cells were re-seeded in 10 cm dishes. After 6 days of transfection, puromycin was added 0.5 μg ml^−1^ to cell culture and were further grown for 14 days. Individual puromycin resistant colonies were picked up manually and then expanded in 12-well plates. Initial identification of KO clones was carried out by genomic PCR. Positive clones were further verified by qRT–PCR.

### RNA precipitation

To identify the AK023948-binding partner, we prepared AK023948-biotinylated RNA probe that was used to pulldown assays for whole-cell lysate. To prepare the probe we first introduced T7 promoter in front of the AK023948 by using PCR primers AK023948-T7-5.1 and AK023948-Not1-3.1 (Supplementary Data set 2). The amplified sequence was then cloned into pCR8 vector (Fisher Scientific). The resultant plasmid was then was linearized with NotI and used for *in vitro* RNA synthesis. Total cellular extract was used to mix well with the RNA probe, followed by precipitation using streptavidin agarose beads (Fisher Scientific). BC200 and PCGEM1 served as negative controls in addition to beads alone. The protein attached to beads was separated in PAGE. Silver staining was carried out using Pierce Silver Staining Kit (Fisher Scientific) following their protocol. The band unique to AK023948 was cut out and sent for mass spectrometry analysis (Applied Biomics, Hayward, CA).

### RNA immunoprecipitation

RNA immunoprecipitation was performed using the Magna RIP RNA-Binding Protein Immunoprecipitation Kit (Millipore, Billerica, MA) and different antibodies (p85 and DHX9) according to the manufacturer's protocol. After the RNA was recovered from protein A+G beads, qRT–PCR was performed to detect AK023948.

### *In situ* hybridization

Biotinylated AK0-LNA probe (Supplementary Materials) was used for ISH as previously described^4^. In brief, following pre- and hybridization, and washes, the signal was amplified by TSA amplification kit (Perkin Elmer) and subsequently revealed by Ultra Vision One polymer and AEC chromogen (Fisher Scientifics). The intensity of signal in TMA was classified as − (negative),+(weak positive) and ++ (strong positive). To make sure that ISH is specific to AK023948, we designed a blocker which is a complementary sequence of AK0-LNA probe. We used 10 times higher concentration of blocker than AK0-LNA during hybridization. Procedure for fluorescence *in situ* hybridization was essentially same as above except that signals were revealed by TSA Kit #24 with Alexa Fluor 568 (Life Technology).

### Immunohistochemistry

To detect pAKT in clinical samples we re-used the TMA, which was previously used to detect AK023948 by ISH. We treated the TMA in 1% acid alcohol (HCl+ethanol) for 1 min to remove the horseradish peroxidase (HRP) signal from ISH^23^,^24^. After washing, the slide was boiled in 10 mM sodium citrate at pH 6 and treated with 3% H_2_O_2_, and then blocked in 3% BSA. Finally, the slide was probed with pAKT antibody, using ultravision one detection system HRP polymer and AEC chromogen (Fisher Scientific) following the manufacturer's protocol. The clinical correlation between pAKT and AK023498 expression was determined by Fisher's exact test for association.

### Co-immunoprecipitation

Corresponding antibody or IgG was added to cell lysate that was treated with A+G agarose beads (Fisher Scientific). The mixture was rotated for 1.5 h and precipitated beads were washed three times with PBS containing protease inhibitor cocktail. The protein lysates were separated in SDS–PAGE.

### Proximity ligation assay

PLA was carried out using Duolink *in situ* fluorescence kit (#DUO92101 from Sigma) according to the manufacturer's protocol. In brief, HeLa cells were simultaneously transfected with Myc-p85 plus control siRNA or AK0 siRNA in culture chambers and 48 h later, the cells were fixed with 4% paraformaldehyde and permeabilized with 0.1% Triton X, followed by 3% BSA for blockage. Myc tag (mouse) and DHX9 (Rabbit) antibody were added and incubated at 4 °C overnight. Secondary antibodies conjugated with oligonucleotides (Rabbit antibody with PLA probe plus and Mouse antibody with PLA probe minus) were incubated for 1 h at 37 °C after primary antibody. After wash, ligation was taken place for 30 min at 37 °C, followed by amplification with polymerase for 2 h at 37 °C.

### Animal work

Female nude (nu/nu) mice (4∼5 weeks old) were obtained from Harlan Laboratories (Indianapolis, IN). All animal studies were conducted in accordance with the NIH animal use guidelines and a protocol was approved by the School's Animal Care Committee. MCF-7 AK023948 KO#13 and MCF-7 gRNA vector control cells were harvested at exponential growth stage and mixed with 50% matrigel (BD Biosciences, Franklin Lakes, NJ). One million cells/spot were injected to mammary fat pad of mouse as described earlier^57^. Growth of tumours was monitored and measured every other day after 7 day of tumour cell injection. The volume of tumour was calculated by using formula, *V*=1/2 (width^2^ × length).

### Statistical analysis

Although the researchers conducting the experiments were not blinded to the group allocation, statistician was blinded from group allocation when performing statistical analyses. The continuous outcomes were summarized as the mean and s.e.m. The normality of data was checked by the stem and leaf plot, and the data were approximately normal. The two-sample *t*-test was used to compare the mean of continuous outcome between two experimental conditions. The Satterthwaite *t*-test was used when unequal variances were confirmed by Levene's test. The Bonferroni correction was applied in the experiments involving comparisons at multiple time points or among more than two experimental conditions. The Kaplan–Meier method was used to estimate the survival probability in subgroups determined by expression level of DHX9.

### Data availability

The authors declare that all the data supporting the findings of this study are available within the article and its Supplementary Information files and from the corresponding author upon reasonable request.

## Additional information

**How to cite this article**: Koirala, P. *et al*. LncRNA AK023948 is a positive regulator of AKT. *Nat. Commun.* **8**, 14422 doi: 10.1038/ncomms14422 (2017).

**Publisher's note**: Springer Nature remains neutral with regard to jurisdictional claims in published maps and institutional affiliations.

## Supplementary Material

Supplementary Information Supplementary Figures

Supplementary Data 1 A list of gRNA sequences for SAM library

Supplementary Data 2 A list of primers used in this study

Peer Review File
