# Supplementary material for: LncRNA AK023948 is a positive regulator of AKT
Source: Nat Commun. 2017 Feb 8;8:14422. doi: 10.1038/ncomms14422 (PMC5309785; doi:10.1038/ncomms14422)
Supplement: Supplementary Information — Supplementary Figures [file ncomms14422-s1.pdf]

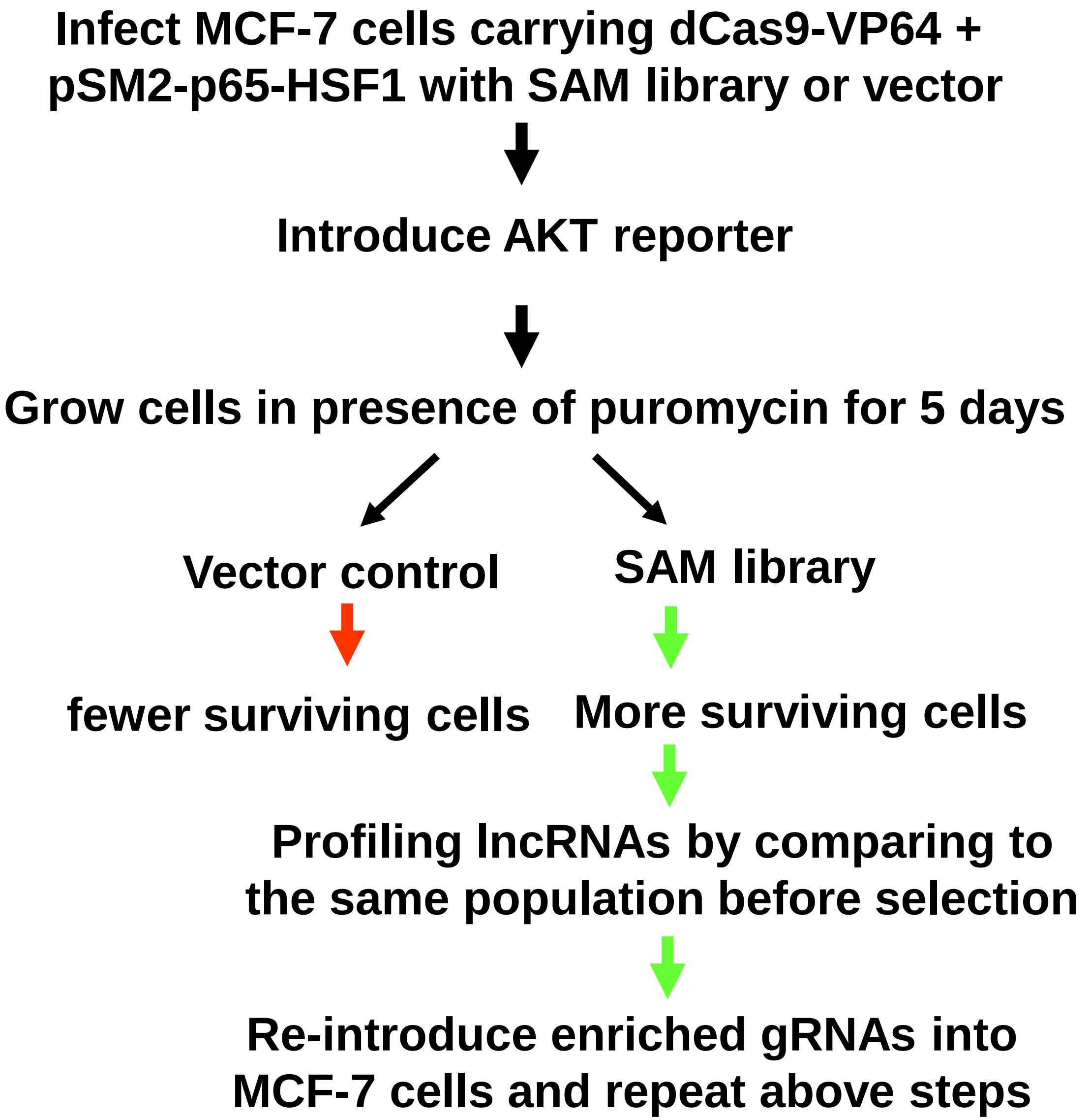

Supplementary figure 1 Screening procedure

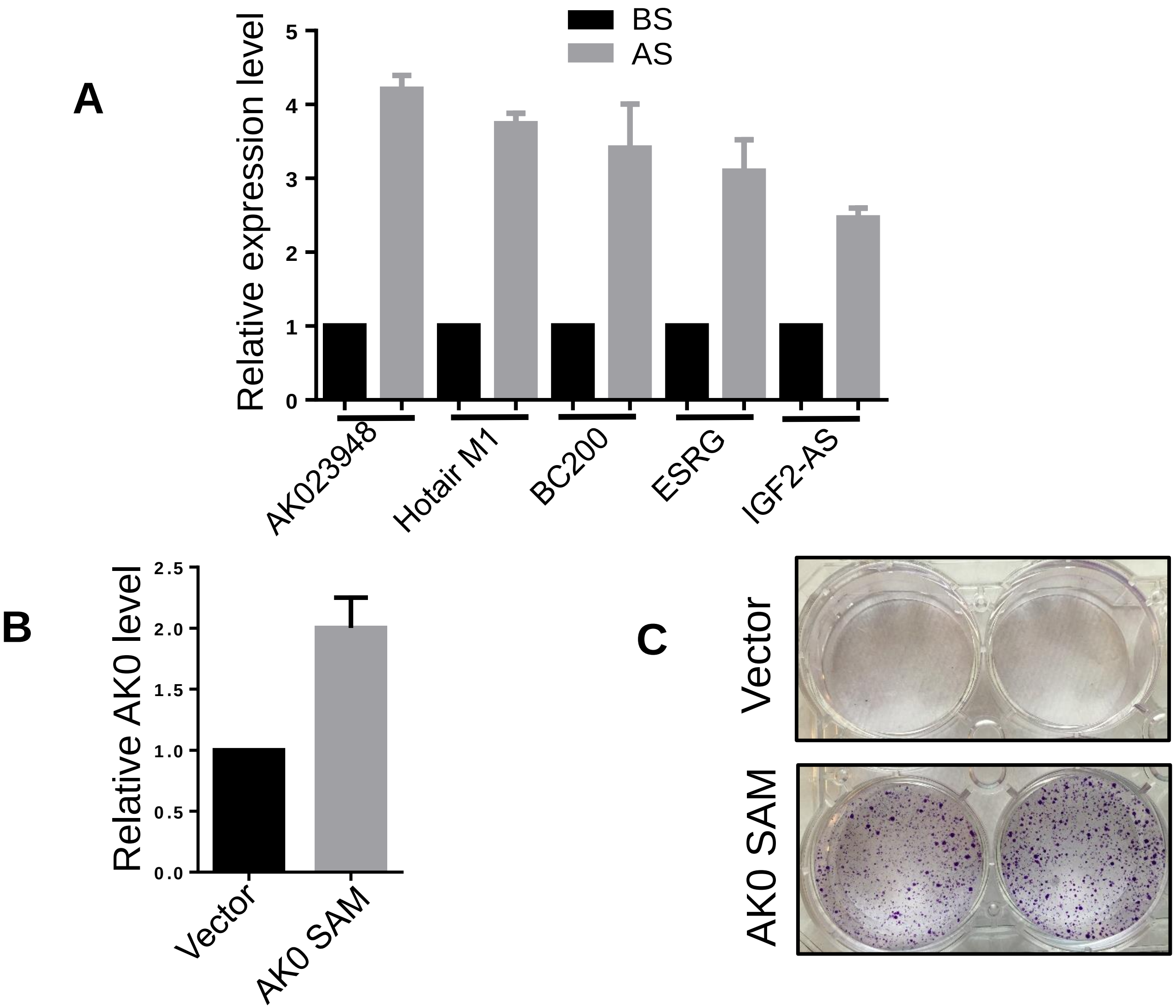

Supplementary figure 2 Identification of potential lncRNA for AKT activation by screening SAM lncRNA library. A, Five lncRNAs were identified as candidates. BS, before selection; AS, after selection. B, AK0 SAM gRNAs increase the endogenous AK0 level. C, AK0 SAM gRNAs confer resistance to puromycin.

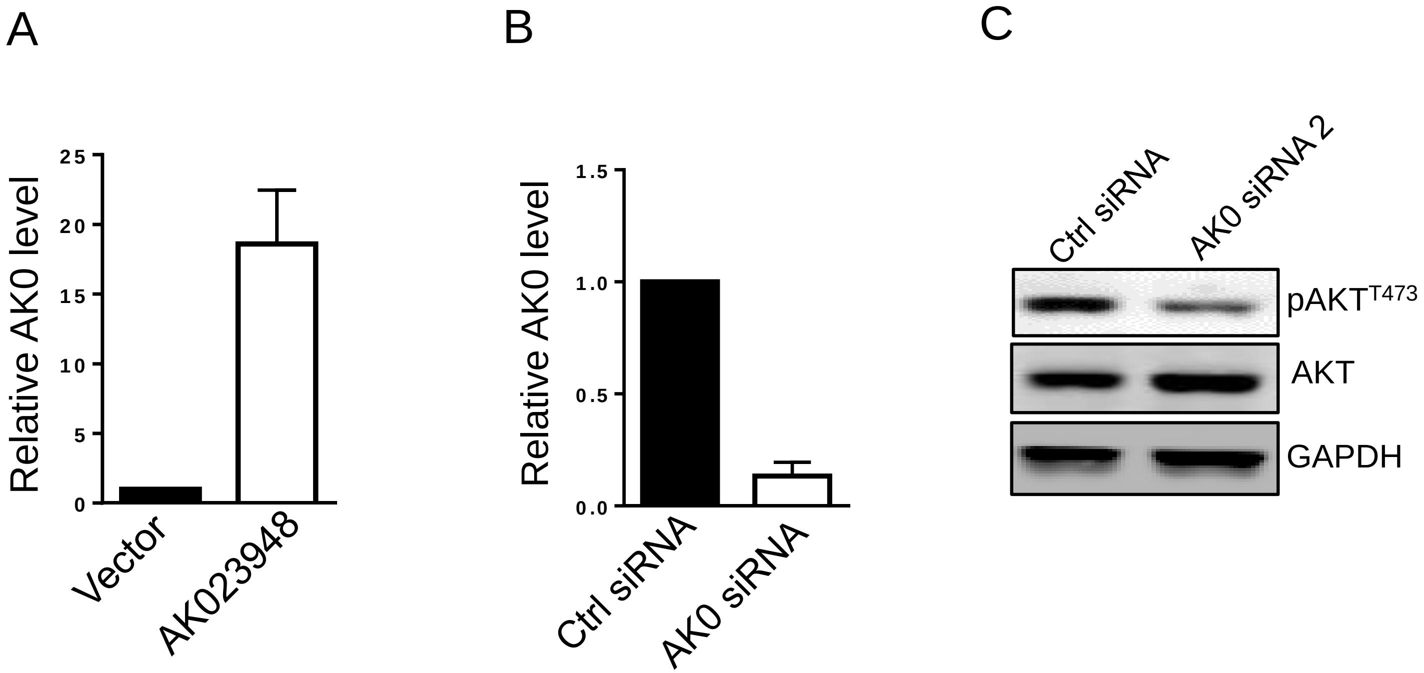

Supplementary figure 3 A & B, Effect of ectopic expression of AK023948 and AK0 siRNA on AK023948 expression, as determined by qRT-PCR. C, AK0 siRNA2 also suppresses AKT activity.

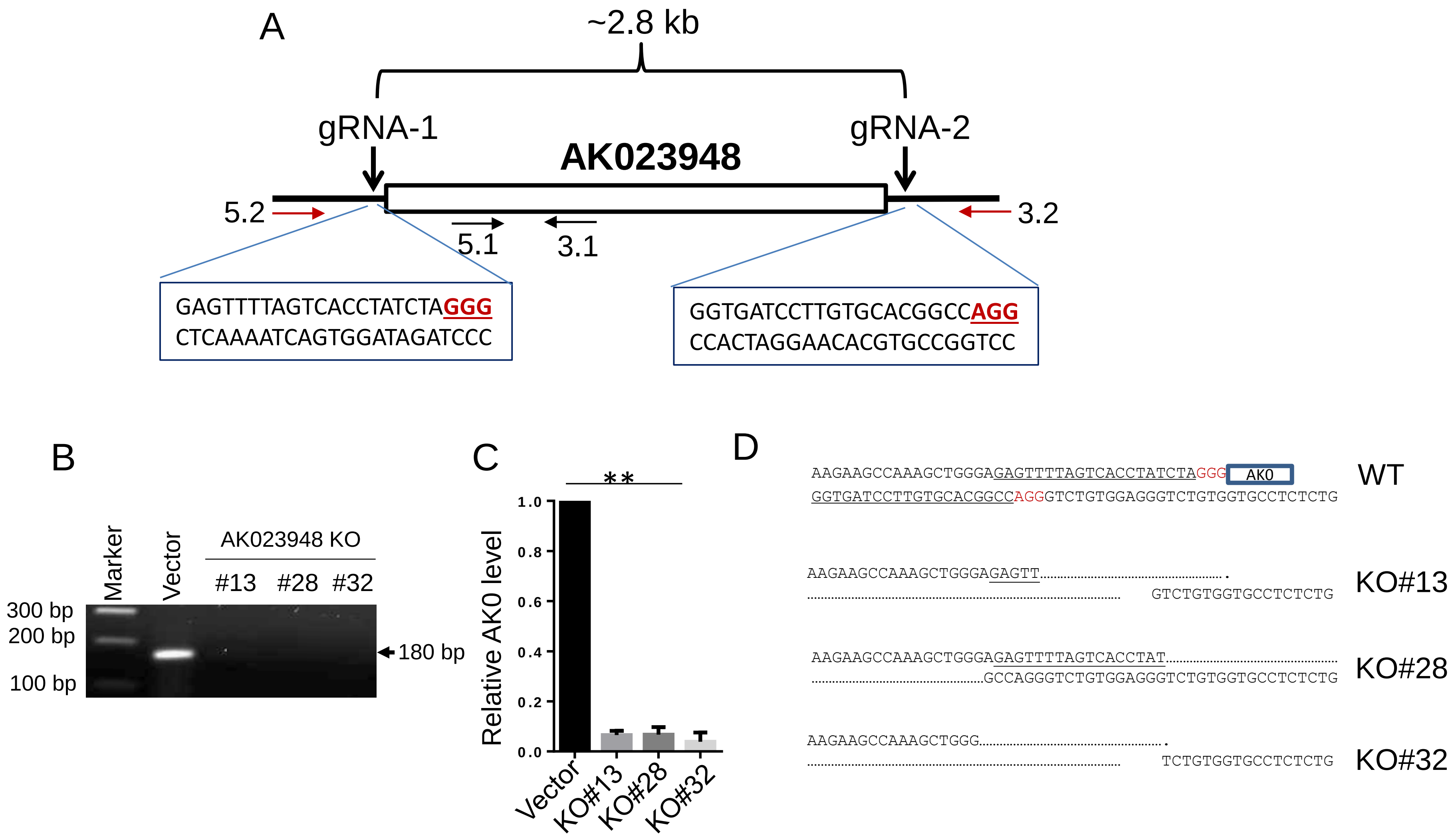

Supplementary figure 4 Generation of AK023948 knockout by CRISPR/Cas9. A, Knockout strategy with gRNA sequences. Relative positions of primers (5.1/3.1) used for genomic PCR and qRT-PCR, and those used to detect deletions (5.2/3.2) are shown under AK0 sequence. B, Identification of KO clones by genomic PCR. C, Detection of AK023948 in KO clones by qRT-PCR. D, Nucleotide sequences of three clones involving deletions.

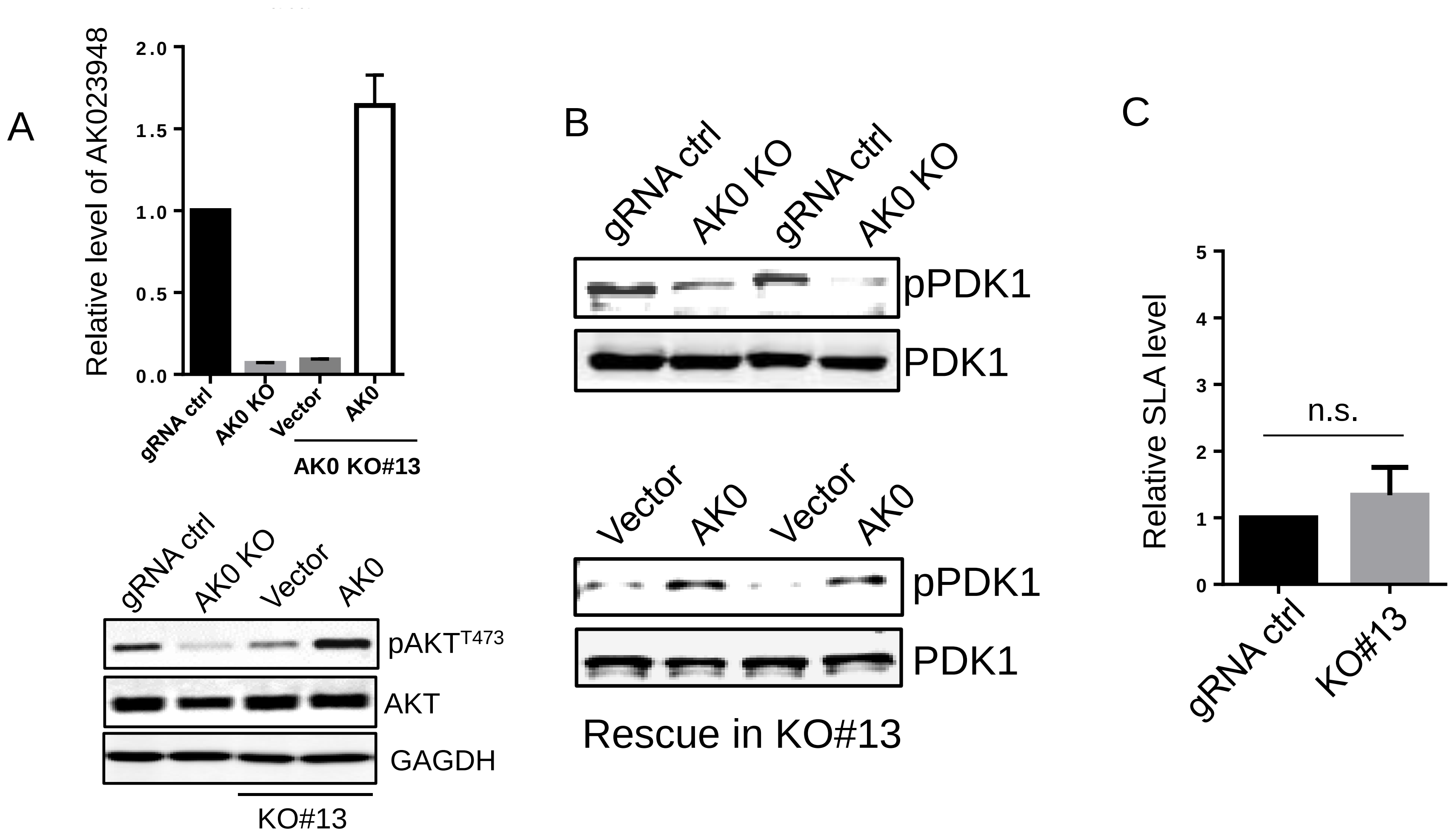

Supplementary figure 5 A, Relative expression of AK023948 after KO (#13) or rescue (top) and its effect on AKT activity (bottom). B, AK0 KO (#13) suppresses PDK1 activity (top) whereas re-expression of AK023948 restores PDK1 activity (bottom). C, AK023948 KO (#13) has no effect on SLA.

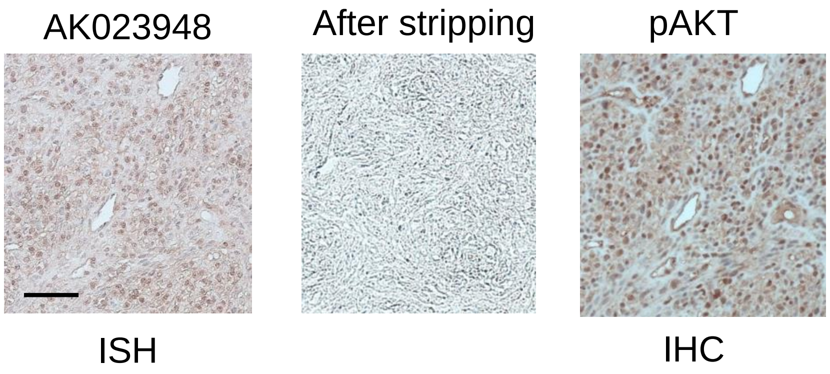

Supplementary figure 6 Sequential detection of AK023948 and pAKT in the same breast tumor tissue by ISH and IHC, respectively. After ISH, the tissue was treated with 1% acid alcohol to remove the ISH, followed by IHC. Scale bar, 100 μm.

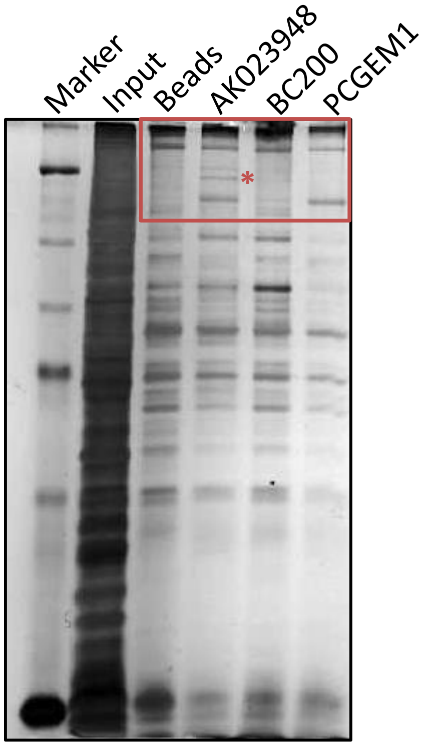

Supplementary figure 7 Analysis of RNA precipitation with PAGE and silver staining. A unique band (*) for AK023948 was clearly visible.

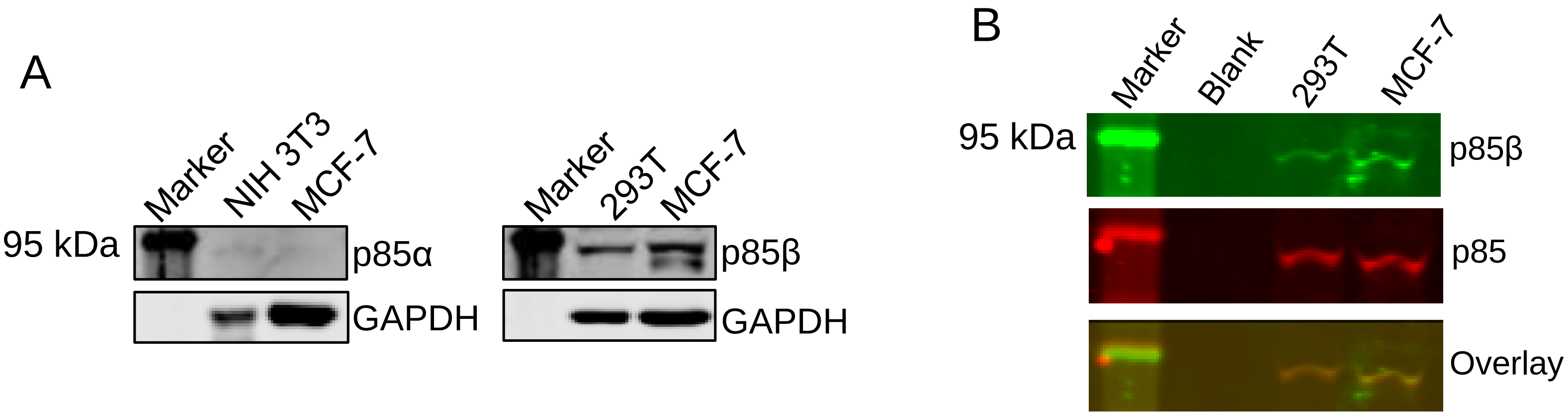

Supplementary figure 8 A, Although there are two major p85 isoforms, we detected a predominant p85β band with p85β antibody, but little p85α with p85α antibody in MCF-7 cells. B, Pan-p85 antibody (p85) recognizes the same band that was recognized by p85β antibody. No p85α was visible for pan-p85 antibody.

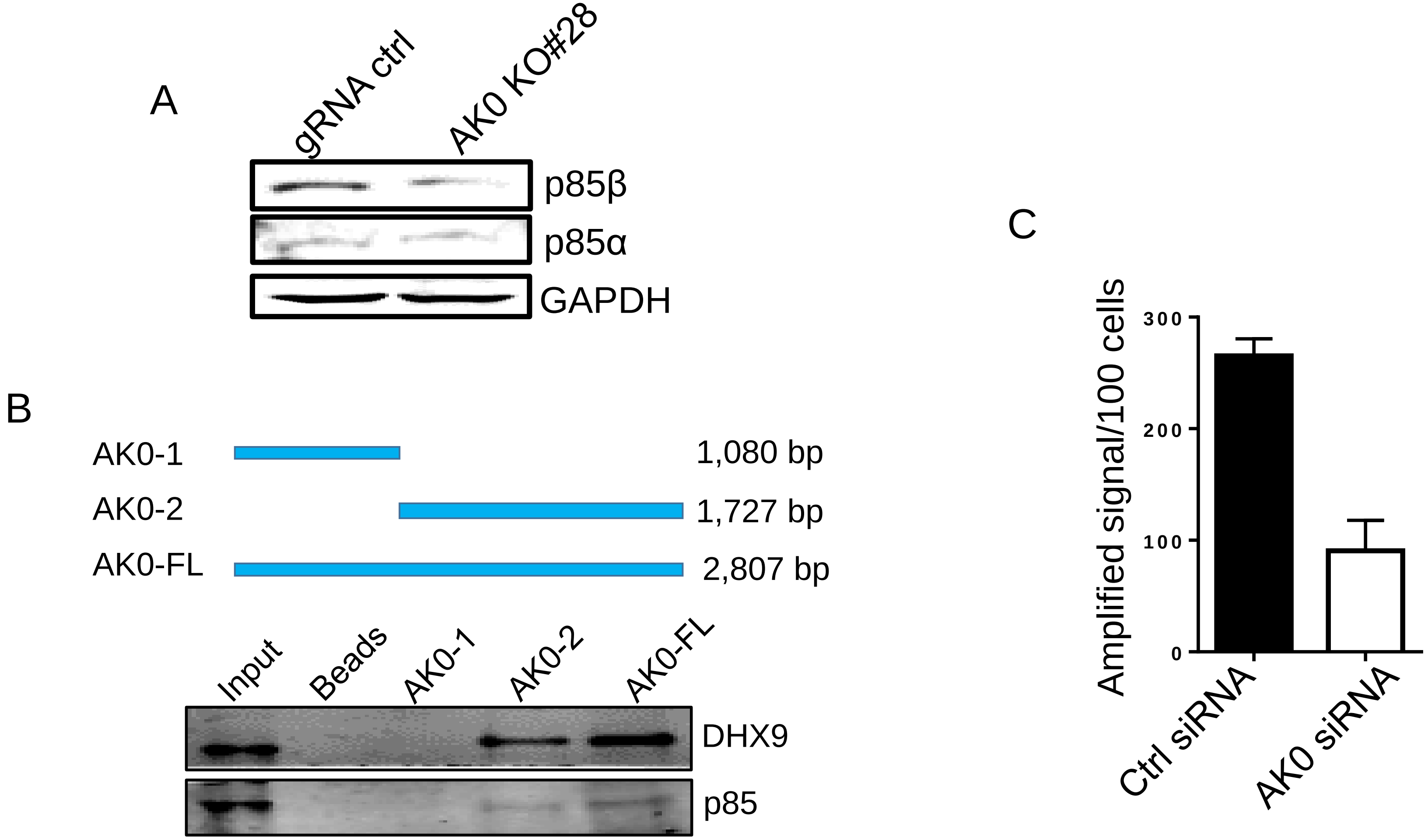

Supplementary figure 9 A, AK023948 KO (#28) suppresses the p85β level, as detected by Western blot. B, Interaction between AK023948 with DHX9 and p85 by RNA precipitation, followed by Western blot. Probes used for precipitation were shown on top; Western blot of the precipitates was shown at bottom. Both DHX9 and p85 interact with AK023948 at 3’ region (AK0-2). C, AK0 siRNA suppresses the interaction between DHX9 and p85, as detected by PLA assay. The number of red spots was counted based on 100 cells each for control siRNA and AK0 siRNA.

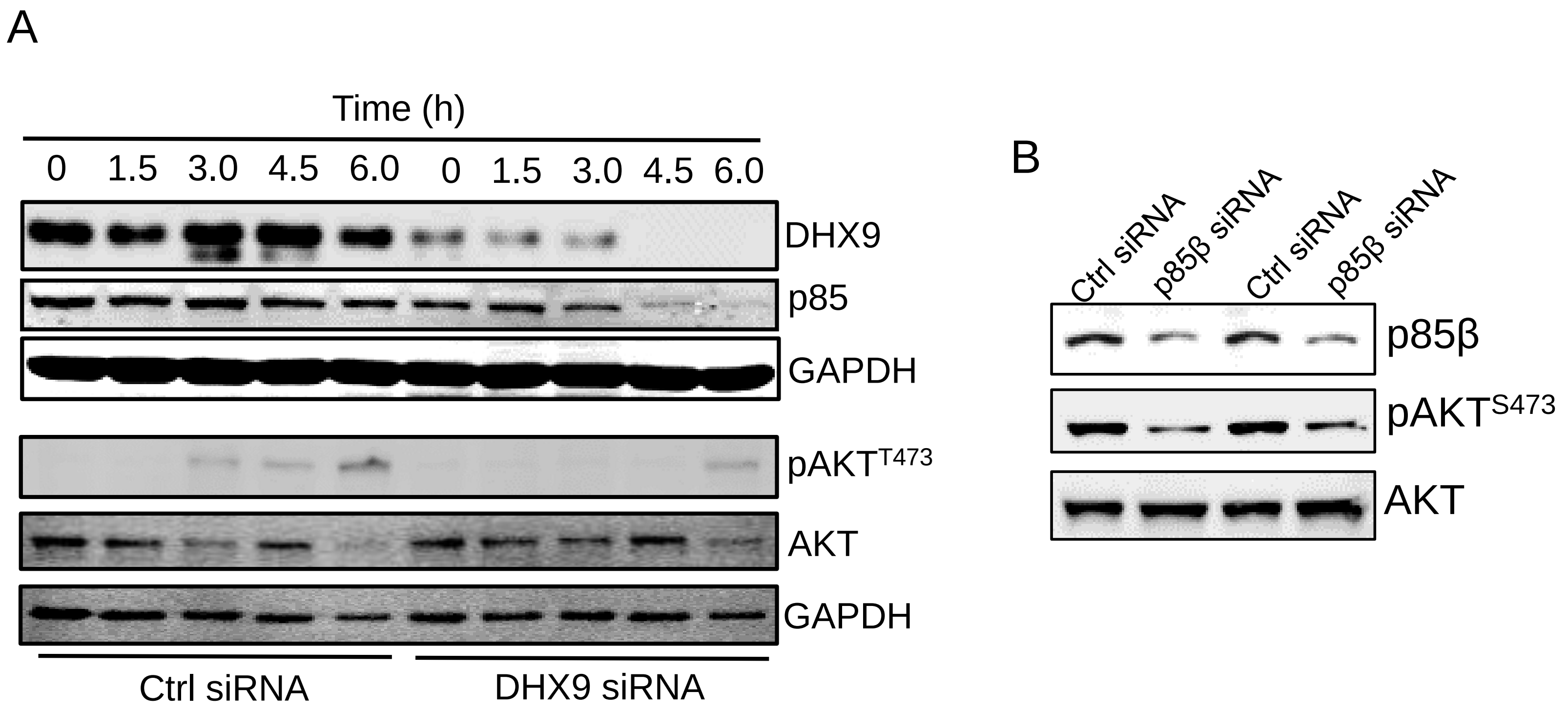

Supplementary figure 10 A, DHX9 siRNAs reduces the p85 stability. Cells were first transfected with DHX9 siRNA and 24 h later CHX was added, and the cells were harvested at indicated time points after CHX. B, p85β siRNA suppresses AKT activity. Two transfection experiments were done on separate days.

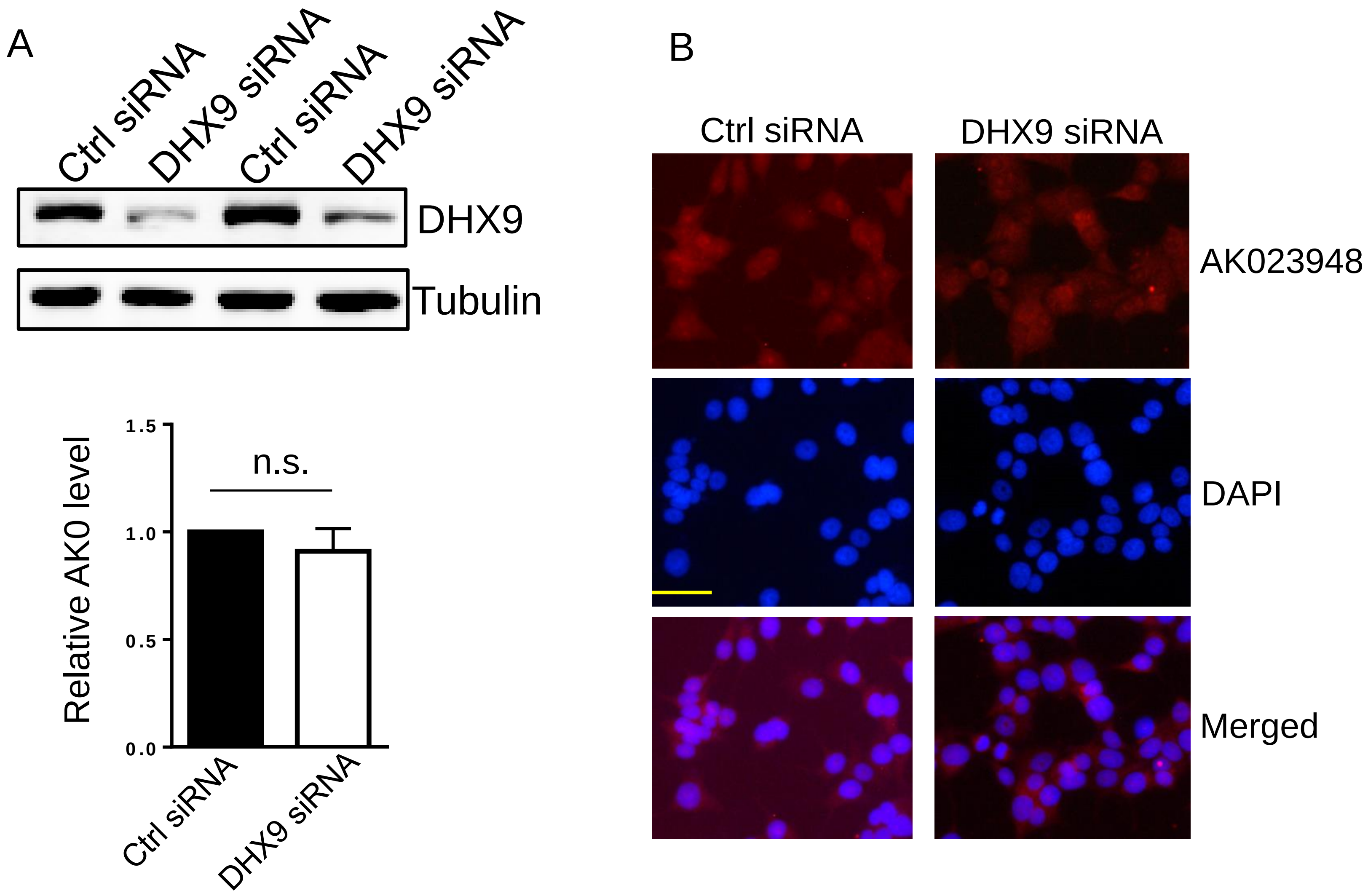

Supplementary figure 11 A, Suppression of AK023948 by DHX9 siRNAs in MCF-7 cells and detection of AK023948 level after treatment with DHX9 siRNAs in MCF-7 cells, by qRT-PCR. Two transfection experiments were done on separate days. B, DHX9 siRNA has no effect on the subcellular localization of AK023948 (by FISH) in MCF-7 cells. Scale bar, 100 μm.

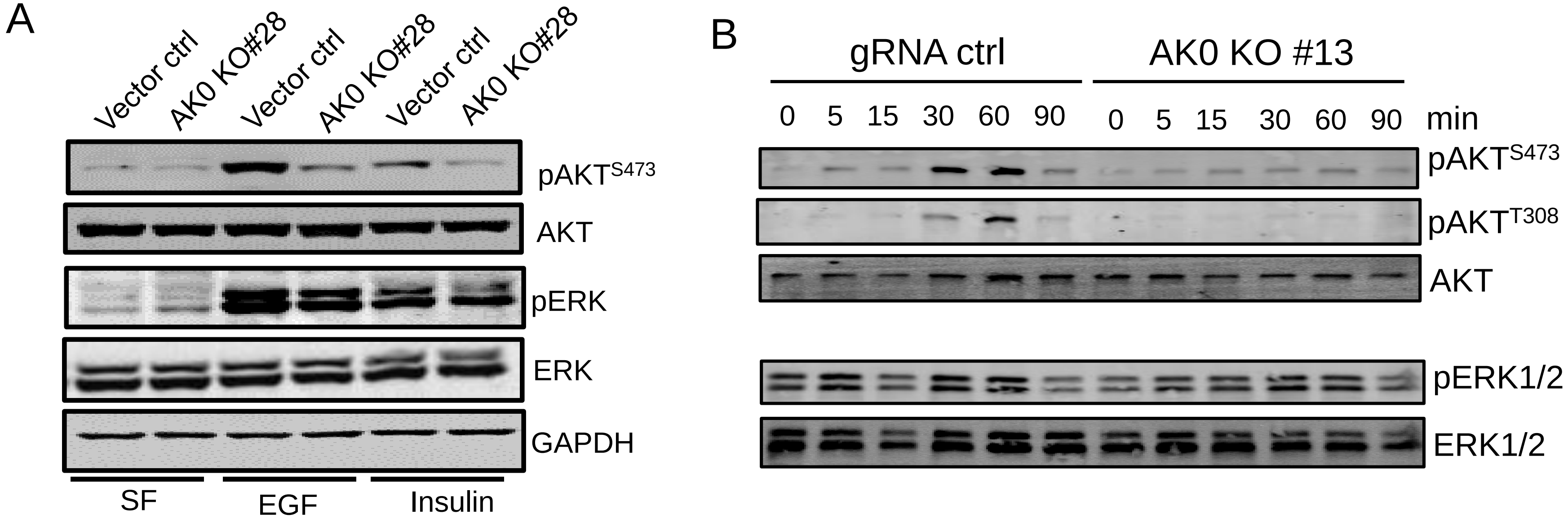

Supplementary figure 12 A, AK023948 KO (#28) inhibits the EGF/insulin-induced AKT activation. B, AK023948 KO (#13) inhibits the insulin-induced AKT activation. Cells were first cultured in serum free medium for 12 h and then insulin was added at 10 ng/ml for indicated time points. Note that little effect was seen for pERK1/2 under the same condition.

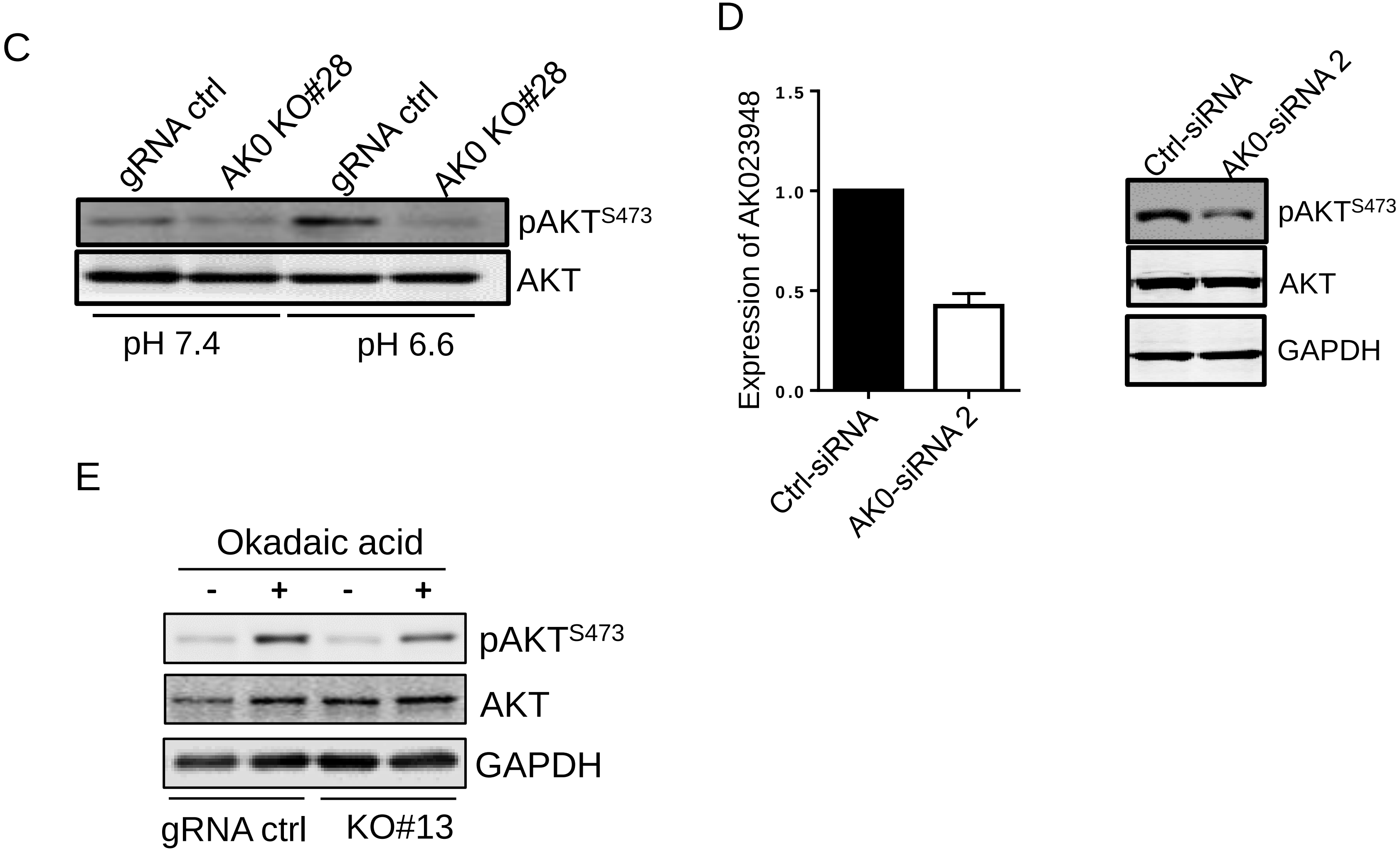

Supplementary figure 12 continued C, AK023948 KO (#28) inhibits the acidosis-induced AKT activation. Cells were cultured at pH 7.4 or pH 6.6 for 2 h before harvesting for Western blot. D, AK0 siRNA2 suppresses pAKT in BT549 cells. E, Suppression of PP2A by okadaic acid increases AKT activity, but this induction is lower in KO #13 than in gRNA control cells. The cells were treated with 0.4 μM okadaic acid for 1 h before harvesting for Western.

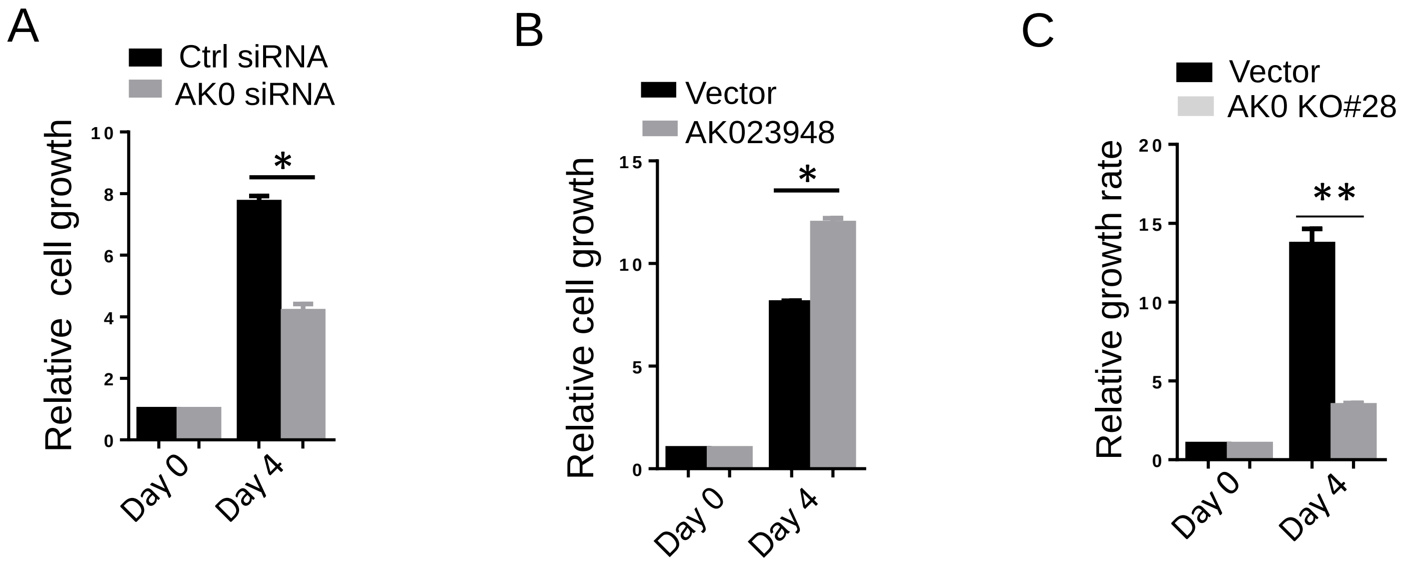

Supplementary figure 13 AK023948 promotes cell proliferation in vitro. A, AK023948 siRNA suppresses cell growth in MCF-7 cells. B, Ectopic expression of AK023948 promotes cell growth. C, AK023948 KO (#28) suppresses cell growth.

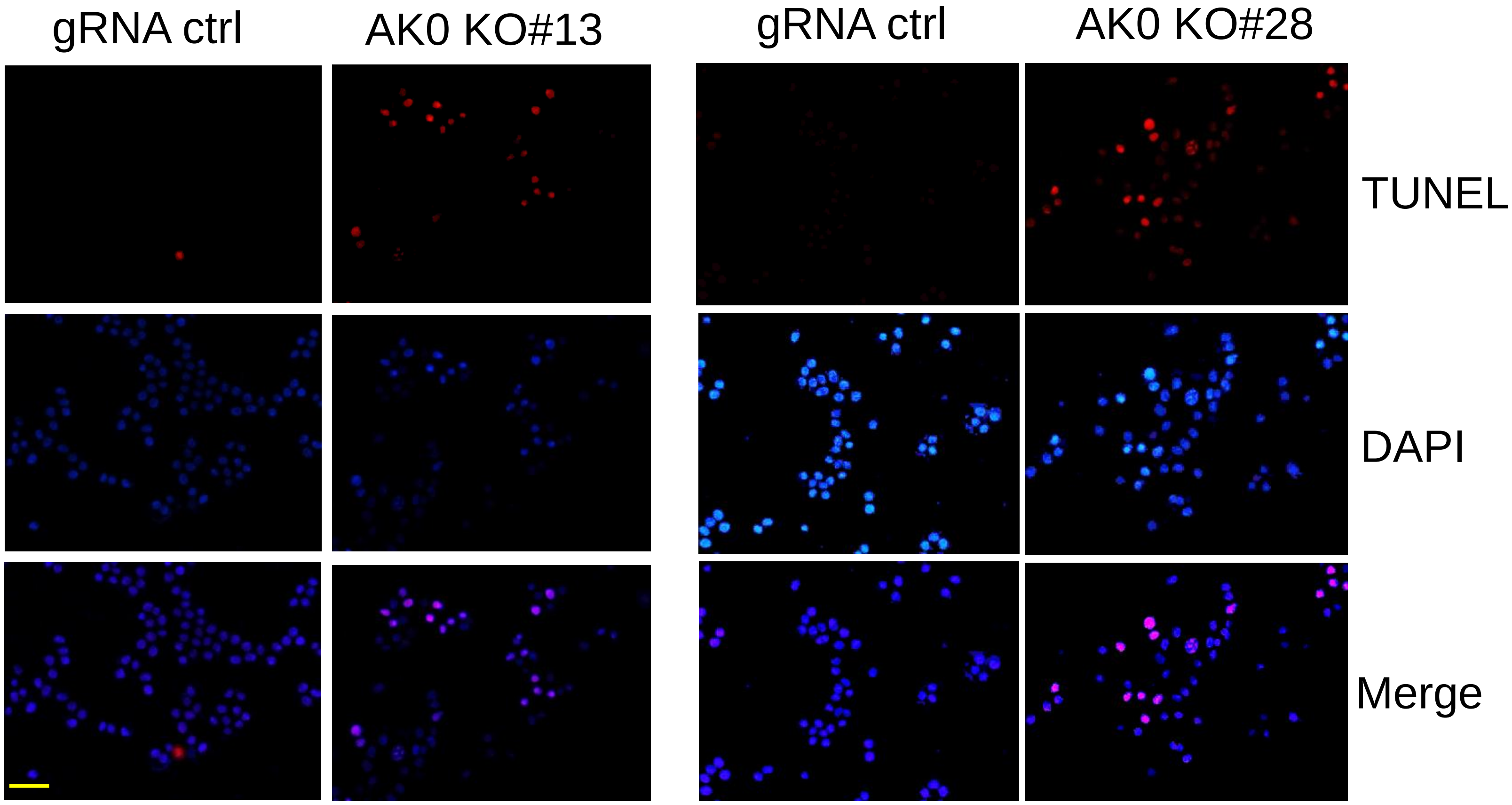

Supplementary figure 14 AK0 KO (#13 and #28) promotes H_20_2-induced apoptosis. Cells were seeded in slide chambers, treated with H_20_2 at 0.8 mM for 4 h before TUNEL assay. Scale bar, 100 μm.

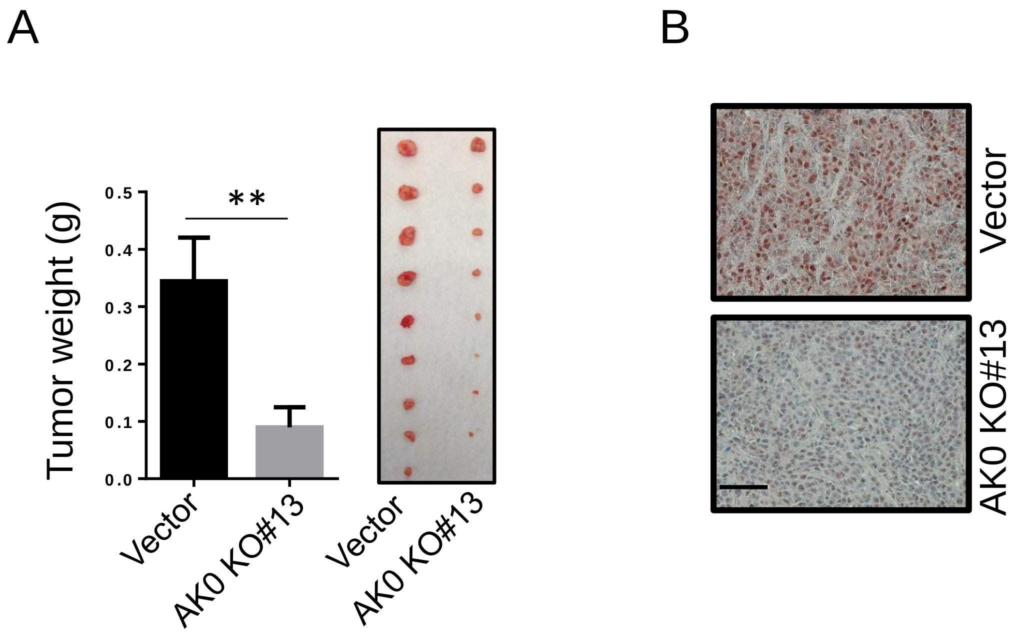

Supplementary figure 15 A, AK023948 KO (#13) decreases tumor weight. B, Detection of a low level of Ki-67 in tumors derived from AK0 KO (#13) cells compared to vector control. Scale bar, 100 μm.

A

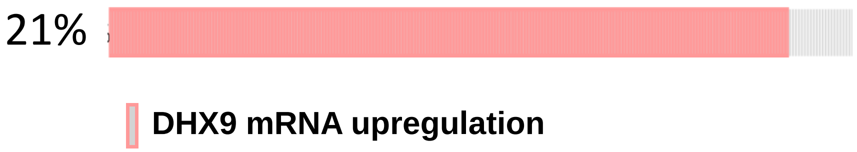

Cases	# total cases	# cases deceased	Median months survival
With DHX9 upregulation	206	36	83.25
Without DHX9 upregulation	695	81	114.72

B

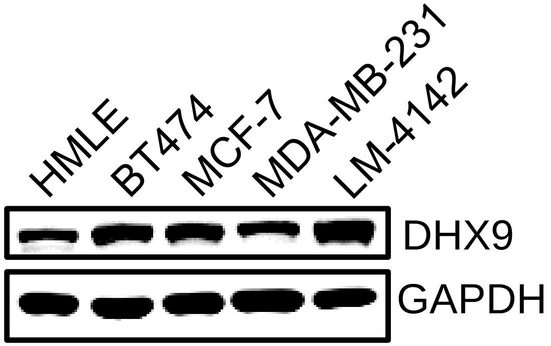

Supplementary figure 16 A, Upregulation of DHX9 can predict overall patient survival. A total of 1091 samples with RNA-Seq data were analyzed for DHX9 expression using QQL (EXP > 1.5). B, Upregulation of DHX9 in breast cancer cell lines as compared to non-malignant HMLE cells.

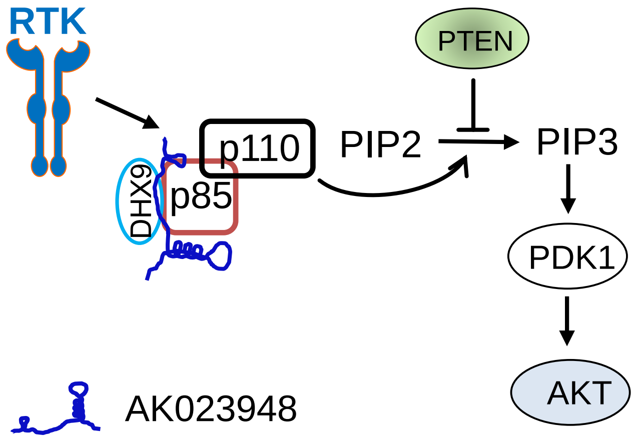

Supplementary figure 17 A working model for AK023948-mediated AKT activation. See explanation in Discussion.

Figure 1B

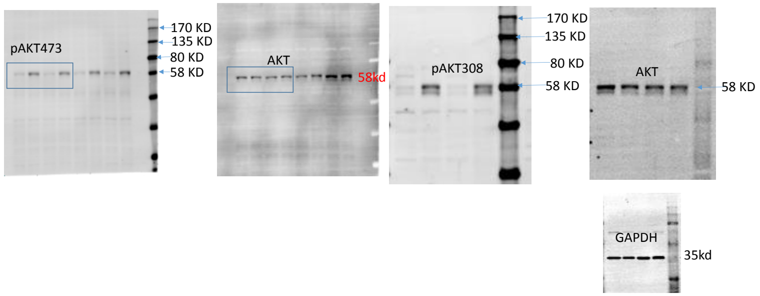

Figure 1C

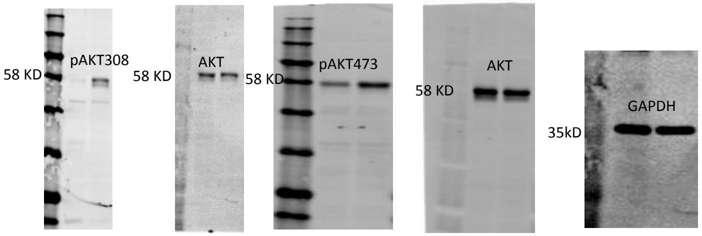

Figure2

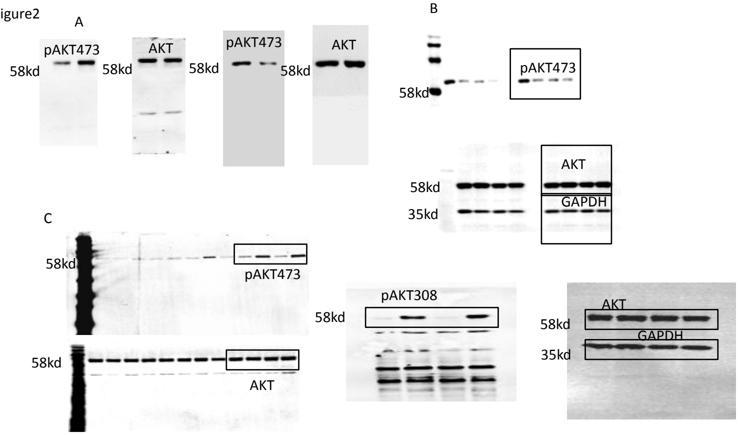

Figure 3 B

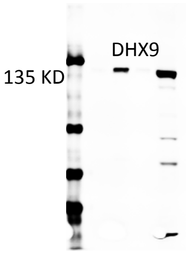

Figure 3 D

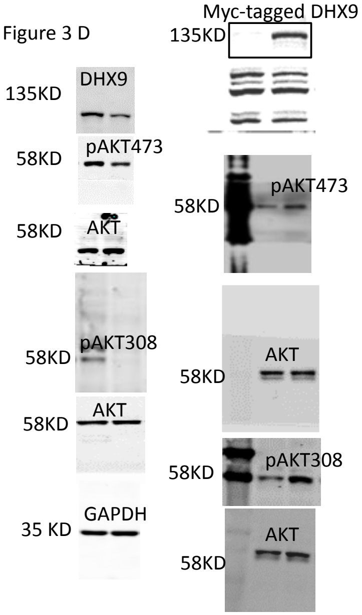

Supplementary figure 18 Uncropped Western blots

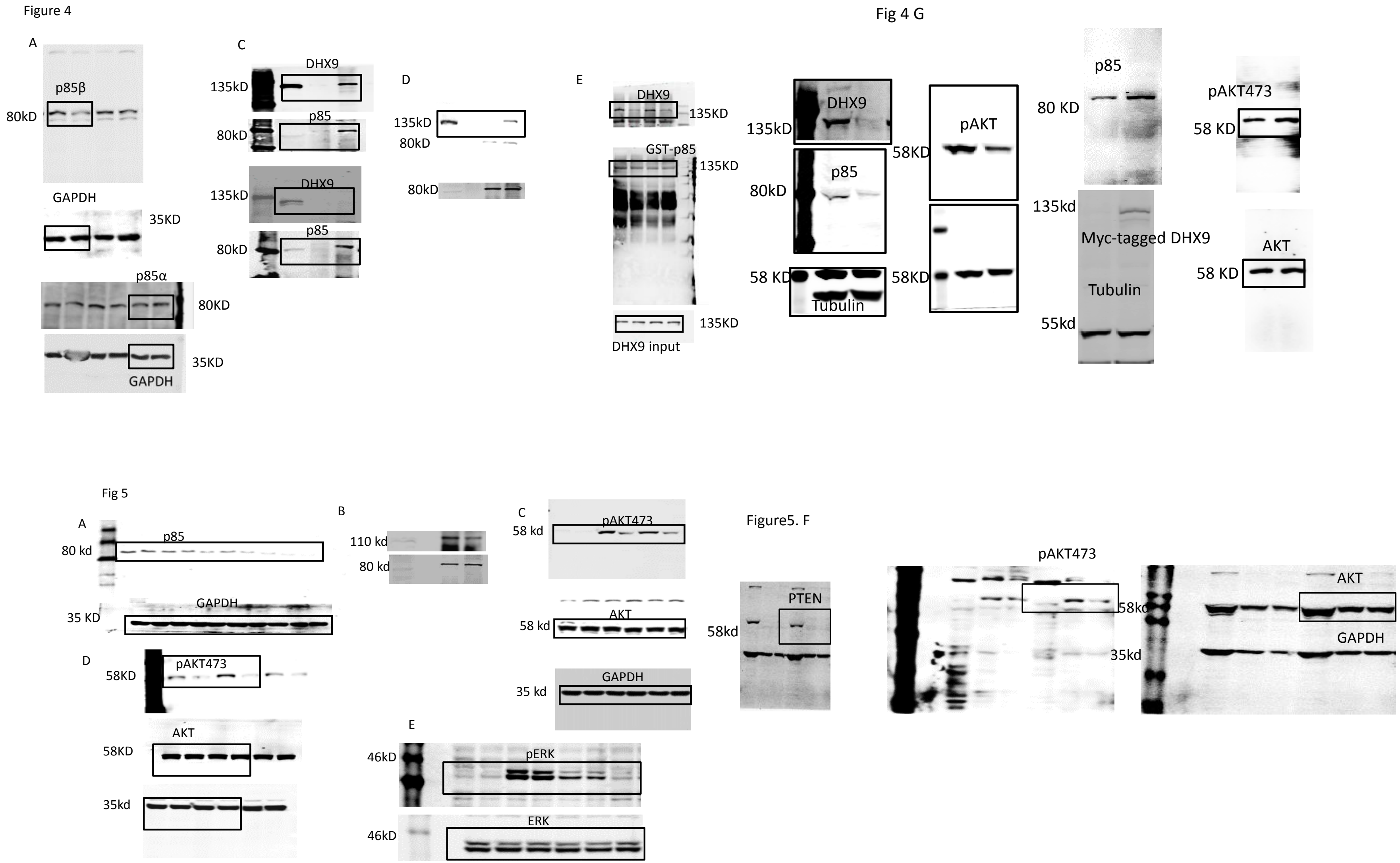

Supplementary figure 18 continued Uncropped Western blots

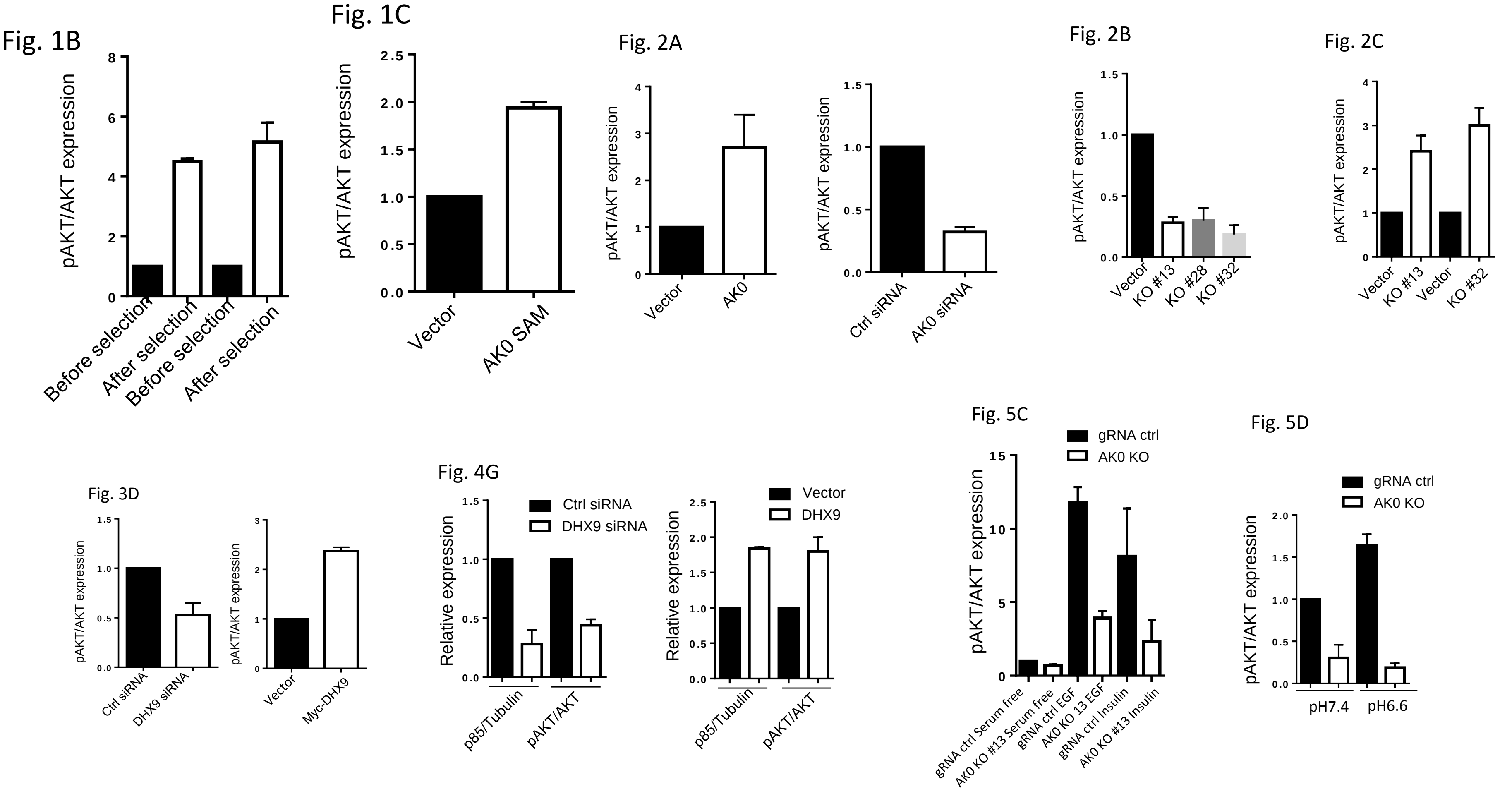

Supplementary figure 19 Quantification of western blots
