# Supplementary material for: LncRNA AK023948 is a positive regulator of AKT
Source: Nat Commun. 2017 Feb 8;8:14422. doi: 10.1038/ncomms14422 (PMC5309785; doi:10.1038/ncomms14422)
Supplement: Supplementary Data 2 — A list of primers used in this study [file ncomms14422-s3.docx]

**Supplementary Table 2, Primers used in this study**

SAM gRNA-5.1 tttcttggctttatatatcttGTGGAAAGGACGAAACACC

SAM gRNA-3.1 CATGTTggccaagttgataacggactagccttattttaac

AK023948-T7-5.1 TAATACGACTCACTATAGGGtttgtcacttccagtgccct

AK023948-T7-5.2 TAATACGACTCACTATAGGGgaagctgagtgccaggtttc

AK023948-Not1-3.1 GCGGCCGCgttttgctaaactaagattt

AK023948-Not1-3.2 GCGGCCGCctaggtgtgtgctcttgggc

AK023948-R1-5.1 ttCtagagctagcgaattcgaagctgagtgccaggtttc

AK023948-RT-5.1 cccacaaagctcttttctgc

AK023948-RT-3.1 ggtgcccaagtaaagcacat

Ak023948-5.2 tcagtcactcccacttataa

Ak023948-3.2 TTAGGCCAATGTGCAATTAG

AK023948-gRNA1 Gagttttagtcacctatcta

AK023948-gRNA2 GGTGATCCTTGTGCACGGCC

AK023948-right-R1-5.1 TTATACGAAGTTATGAATTCGGTGCCTCTCTGGCACTGAA

AK023948-right-R1-3.1 ATAAGCTTGATATCGAATTCAGGTGGAACAGAGCATGTGC

AK023948-left-BamH1-5.1 GCTCTAGAACTAGTGGATCCtctactcctagaactcctta

AK023948-left-BamH1-3.1 GCTATACGAAGTAGGGATCCCgactagttttattagacag

DHX9-Myc-R1-5.1 CCATGGAGGCCCGAATTCtgggtgacgttaaaaatttt

DHX9-Not1-3.1 tcgcagatccttGCGGCCGCttaatagccgccacctcctc

DHX9-RT-5.1 cccacatgacaccagagatg

DHX9-RT-3.1 ttcgtcccaaaggagtcaac

pGEX-2T-BamH1-PI3KR2-5.1 atctggttccgcgtggatccgcgggccctgagggcttcca

pGEX-2T-R1-PI3KR2-3.1 cagtcagtcacgatgaattcgggcggcaggcggcgggccg

PI3KR2-R1-Myc-5.1 CCATGGAGGCCCGAATTCtggcgggccctgagggcttcc

PI3KR2-Not1-3.1 TCGCAGATCCTTGCGGCCGCtcagcgggcggcaggcggc

SLA-RT-E3&4-5.1 ccccagggaaaaagaaagaa

SLA-RT-E3&4-3.1 gaaatcacacgcagtttctcc

TG-RT-5.1 gagaaccaaaatgccctcaa

TG-RT-3.1 agcttccttctgtcgtgcat

AK023948-siRNA-1 GCACAGAACAGAAGCAAGAdTdT

AK023948-siRNA-2 AUAAGAAGAGAAGGAACAAdTdT

AK023948-LNA probe /5Biosg/g+a+g+actgaccctg+a+aaagactcaa+g+g+t

AK023948-SAM-1-5.1 CACCgGCATCACTGTGTAGTGGCGG

AK023948-SAM-1-3.1 aaacCCGCCACTACACAGTGATGCc

AK023948-SAM-2-5.1 CACCgGGATGTAAGCCAGTTCTGAT

AK023948-SAM-2-3.1 aaacATCAGAACTGGCTTACATCCc

AK023948-SAM-3-5.1 CACCgTGCCTCATAGGATTGAGTGA

AK023948-SAM-3-3.1 aaacTCACTCAATCCTATGAGGCAc

AK023948-SAM-4-5.1 CACCgAGGGACCCAGGCAGGCCTTA

AK023948-SAM-4-3.1 aaacTAAGGCCTGCCTGGGTCCCTc

AK023948-SAM-5-5.1 CACCgGGGACTTAGTCAAGAGAGGA

AK023948-SAM-5-3.1 aaacTCCTCTCTTGACTAAGTCCCc

AKO-polyT adaptor-5.1 GACTCGAGTCGACATCGATTTTTTTTTTTTTT

AKO-adaptor-5.1 GACTCGAGTCGACATCGA

AK0-5Race-3.2 tctgtgtgattggttagttg

AK0-3Race-5.1 aagtcacacagcttccacgg
