# Supplementary material for: LncRNA AK023948 is a positive regulator of AKT
Source: Nat Commun. 2017 Feb 8;8:14422. doi: 10.1038/ncomms14422 (PMC5309785; doi:10.1038/ncomms14422)
Supplement: Peer Review File [file ncomms14422-s4.pdf]

Reviewers' comments:

Reviewer #1 (Remarks to the Author): Expert in CRISPR screenings

The manuscript "LncRNA AK023948 is a positive regulator of AKT" presents interesting data relating to novel mechanisms that influence AKT signaling responses. The authors use a pooled CRISPR/Cas9-based synergistic activation mediator screen to identify lncRNAs capable of regulating FOXO-mediated responses in cells. They identify a single lncRNA that binds to AKT and enhances AKT responses under basal conditions and upon growth factor stimulation. Further, the authors identify the DHX9 protein in the AKT/lncRNA complex, suggesting it as a novel regulator of AKT signaling. Overall, the paper makes some very interesting and novel observations regarding AKT signaling. This paper deserves publication in some form as it is also the first to describe a CRISPR-based activation screen using a lncRNA library. Furthermore, there are not many publications to use CRISPR genome editing for lncRNAs. However, there are serious concerns about the description of methodology that makes it very difficult to properly evaluate the quality of data. In particular, the methods are not described in sufficient detail. In addition, some aspects of the proposed model are not fully supported by the data.
Specific comments:
1. The Materials and Methods section lacks sufficient detail about the experiments. Most often, the authors refer to "according to manufacturer's instructions" or to the supplementary materials. But then again, there is not enough detail in the supplementary material section and again, referring mostly to manufacturer's protocols. Examples that are omitted include: which vectors were used, which cells (e.g. in the screen), the methods for identification of screen hits are not described properly (RNAseq?), mass spectrometry is not described, antibodies are not identified (e.g. catalogue or clone number is missing), CRISPR knockout generation is not described, the buffers used for protein isolation are missing, lentiviral transduction is not described. It is often not even clear which cells have been used.
2. Similarly, the Figure legends are very minimalistic and not of appropriate standard. This is a problem for all Figures throughout.
3. Figure 1A doesn't show much of "design". As it stands, the Figure does not show anything informative.
4. Figure 2D: it is not described what the top and bottom panels are.
5. Figure 3B,C: is C the quantification of B?
6. Legend for Figure 6 is not correct. (E) may be (D) and (F) is not described or wrongly labelled? What is meant by Figure 6(C) "bottom"? This is very confusing.
7. There is no validation of the CRISPR Knockouts. RT-PCR is mentioned in Figure S3. It would be good to know where the primers bind to, maybe shown in a scheme. The problem with CRISPR-mediated knockout of lncRNA is that validation has to be done at the RNA level. The authors are assuming that the dual gRNA design will result in a complete deletion of the entire transcript. However, this is not necessarily the case. One needs to evaluate whether both guides were efficiently processed. Also, whether both alleles (or multiple alleles, depending on the cell type - it would help to know which cells were used) are modified. It is even possible that there are only local insertion/deletions rather than loss of the entire locus. All this information will be useful for other researchers who plan to generate lncRNA knockouts. For this, each allele for each clone needs to be sequenced at the nucleotide level surrounding the gRNA target sites.
8. The model presented in Figure 5F is not supported by the data. If the authors want to claim that AK023948 is required for binding of p85b to DHX9, this would need to be done with purified components in vitro. But then, Figure 5F is not cited in the text nor discussed.
9. The claim that p85alpha does not bind to the lncRNA is not fully supported. For instance, in Figure S6 the statement "No p85alpha was visible for pan-p85 antibody" is not backed by any data.
Minor points:
1. page 5: "cell singling" should be corrected.
2. page 8, top: "... together they regulates..." should be "regulate".
3. Page 8, top: "detectible" should be "detectable".

4. page 10: "In consistent..." should be "In consistence...".

Reviewer #2 (Remarks to the Author): Expert in lncRNAs

The present manuscript details a screen in which a number of lncRNAs were tested for the ability to positively regulate AKT. The top hit from the screen, AK023948 (AK0) lncRNA, was selected for further study. Through a combination of loss and gain of function study and physical association studies, the authors show that AK0 interacts with the RNA helicase DHX9, and that the stability of the oncogenic isoform of the regulatory subunit of PI3K, p85b, is compromised by the loss of function of either the lncRNA or the helicase. This, in turn, affected the activation of AKT through the PI3K axis. The presented data are interesting and novel, and the experimental approach taken are mostly appropriate. Further, the quality of the presented data is high and they are presented in a clear manner, except in Fig. 6 when the text and the figures do not match (e.g. Fig. 6C is not a tumor array). However, a number of issues have to be addressed to ensure the reproducibility of the data. Further, while the main conclusion of the manuscript, the involvement of AK0 and DHX9 in stability of p85, is well-supported, the interaction network between these three factors is not fleshed out. These issues are detailed below.

1) A major issue is the absence of sufficient controls in loss of function studies to ensure that the observed impacts don't stem from off-target effects in the case of siRNA approaches or clonal effects in the case of CRISPR-mediated KO approach. In all cases, more than one siRNA/KO cell line must be used, or, a rescue experiment must be shown. A number of important experiments in this study unfortunately lack these controls, which should be included.

2) In the rescue/gain of function experiments when AK0 lncRNA or a protein-coding RNA is ectopically expressed, the level of overexpression compared to endogenous levels should be determined.

3) Similarly, in all knock down studies, the extent of knock down should be determined.

4) The physiological importance of the interaction between AK0 and DHX9 is an important unaddressed issue. Do the knock down or overexpression of DHX9 affect the stability or subcellular localization of AK0?

5) Similarly, to understand the mechanism of the observed regulatory impact, the mode of interaction of DHX9 and AK0 with p85 should be better fleshed out. Do they both directly interact with p85, or one alters the conformation of the other so it can interact with p85? An informative experiment in this regard could be mapping the region in AK0 that is needed for interaction with DHX9 and p85. Do these interactions map to different regions of the RNA (suggesting that the lncRNA acts as a scaffold) or the same region (which could mean that AK0 indirectly interacts with p85 through DHX9)?

6) Please correct the text/figure discord in Fig. 6.

Minor points:

1) p3, line 38. lncRNAs are not usually capped and poly-A-ed. A large fraction of lncRNAs are not polyadenylated.

2) In Fig. 2C, does the rescue experiment restore the level of pAKT to the level in naive cells?

3) Page 8, first paragraph. Data in Fig. S6A does not provide any information about the relative abundance of the two isoforms. The difference in the signal intensity cannot be compared for different antibodies or even the same antibody on the same blot, as the affinity of the antibody for the two targets may be different. The same is true for the other estimations of abundance made based on signal intensity of western blots.

Reviewer #3 (Remarks to the Author): Expert in AKT signalling in cancer

AKT is a protein serine/threonine kinase that plays a central role in the regulation of various cellular processes, particularly survival and metabolism. It is activated downstream of PI3K, due to phosphorylation of its Thr308 by PDK1 and Ser473 by mTORC2. Importantly, AKT is often dysregulated in cancer, however, the underlying mechanism is still not fully understood. Here, the authors show that the LncRNA, AK023948, acts as a novel positive regulator of the PI3K/AKT pathway. Moreover, they provide evidence that AK023948 exert its function by interacting the ATP dependent RNA helicase A (RHA/DHX9) and the regulatory subunit of PI3K, p85beta. These interactions stabilize PI3K and therefore can sustain high basal activity of AKT.
Generally, the manuscript is well written, presenting important and interesting new data. However, some of the conclusions drawn regarding the molecular mechanism of action are not fully substantiated and some other points need to be addressed in order to make this study suitable for publication. These are as follows:

1. The authors hypothesize that LncRNA AK023948 exert its function by interacting with DHX9 and p85, which stabilizes the latter. However, it is not clear to me that the small differences in p85 expression are sufficient in order to induce AKT activation. Moreover, it is possible that AK023948 and DHX9 interact with additional upstream components to cause the effects. Therefore, It is suggested to modulate p85beta expression by SiRNA or overexpression in order to verify that the effect is indeed mediated by the stabilization of p85.
2. Moreover, more experiments are required in order to verify the existence/formation of the AK023948/DHX9/p85 heterotrimer. For example, sizing column that will show whether this complex is formed by itself, or whether it interact with additional proteins. In addition, the interaction between the proteins is based on CoIP experiments only. The authors should use additional methods to confirm their point, such as proximity ligation assay (PLA) or FRET.
3. The authors showed that PTEN is not involved in the suppression of pAKT. It would be important to check whether the effect is mediated by PP2A and PHLPP that regulate AKT phosphorylation directly.
4. It is clear from the results that AK023948 affects AKT phosphorylation. However, it was not mentioned what residue was followed (T308 or S473), and whether there was a significant change in the activity of AKT. If the effect was just on T473, it is possible that the actual activity of AKT was not change, and may lead to distinct conclusion regarding the mechanism of AK023948 action.
5. Many of the changes presented in the blots are very small, and it is not clear how significant they are. The authors should quantify their results, and include statistical analysis and significance to all figures.
6. An interesting question that hasn't been addressed is whether the knock out of AK023948 induces cell apoptosis that is usually seen when AKT activity is reduced. This would provide a strong indication on the significance and robustness of the AK023948 effect.
7. The starvation used in Fig. 5 (2 hours) is very problematic, as the change of medium/serum by itself is known to induce rapid ERK and AKT activation, and a later induction of feedback inhibition. It is very likely that the effects here were measured when the negative feedback was maximal. Therefore, the authors should use at least 12 hours starvation (or no starvation at all). In addition, the authors should include more time points (5-90 min) to the EGF and Ins stimulation, as it is

possible that some effects, especially on ERK will be seen at different time points.
8. In Figure S6C, please blot for p-AKT as well.
9. Some of the "signaling terminology" is problematic, and more details should be added to the section on AKT in the introduction.
10. Page 9 line 186- the reference was probably supposed to be towards figure 6E and not 6C as indicated.
11. Page 10 line 194- the reference was probably supposed to be towards figure 6D and not 6E as indicated.
12. I am not an expert in the field of LncRNA, but I found it surprising that the authors did not describe the genomic location of AK023948. Looking at it, I found that AK023948 is all within an intron of two other genes, which is probably problematic for expression, as well as SiRNA or CRISPR targeting. These points should be properly discussed.

Reviewer #4 (Remarks to the Author): Expert in breast cancer

Suggested decision: major revise
Comments:
The manuscript entitled "lncRNA AK023948 is a positive regulator of AKT" identified lncRNA AK023948 as a positive regulator of AKT pathway by using synergistic mediation activator (SAM) and AKT reporter system. Mechanismly, the authors showed AK023948 could bind DHX9 and mediated interaction between DHX9 and p85, which increase the stability of p85. This work provide a linkage between lncRNA and AKT pathway, which will deepen our understanding of lncRNA and AKT pathway. Moreover, the screening methods described in this manuscript is very useful for investigation of roles of lncRNA in other important pathways. But there are some critical issues that should be addressed by the authors:
1. Inverstigation of some basic information of AK023948 shows AK023948 is an intronic lncRNA that located in the intron of TG(thyroglobulin) gene, interestingly it is also in the intron of another gene, Src-like-adaptor (SLA). AK023948 has the same direction with TG and opposite direction of SLA. It is reported that src-like adaptor Protein (SLAP) binds to the receptor tyrosine kinase Flt3 and has impact on FL-induced PI3K-Akt pathway. The authors used CRISPR/Cas9-based method to overexpress or knock out this lncRNA, it is very likely that these genome-editing methods may also influence the host gene, e.g., transcription and/or splicing. The authors should demonstrate CRISPR/Cas9 and siRNAs have no impact on host genes to rule out the possibility that activation of AKT is caused by host gene rather than AK023948. Furthermore, it is suggested the authors should provide subcellular localization and the full-length imformation (using 5'RACE and 3'RACE) of AK023948.
2. Since AK023948 activates AKT is the most important finding in this work, the authors should give more evidence to show AKT pathway is truly influenced by AK023948, e.g., the AKT reporter activity and downstream gene expression. Moreover, the authors did not mention which phosphorylation site of AKT was detected.
3. The authors suggested AK023948 can as a scaffold of DHX9 and p85, but p85 can bind AK023948 was only proved by RIP-qPCR, the authors should use the same RNA precipitate used in Figure 3A and 3B to detect p85 by western bolt. Furthermore, DHX9 co-IP to show DHX9 can interact with p85, since this interaction is very important for AKT activation, the authorsed should use in vitro study like GST-pull down experiments (with or without AK023948) to further demonstrate DHX9 and p85 can interact in vitro and AK023948 is essential for the interaction.
4. In Figure 1, Figure 2, Figure 3 and Figure 5C, total ATK potein is used as control, the authors should use housekeeping gene for loading control to show AK023948 have no impact on total AKT.

Reviewer #1 (Remarks to the Author): Expert in CRISPR screenings

The manuscript "LncRNA AK023948 is a positive regulator of AKT" presents interesting data relating to novel mechanisms that influence AKT signaling responses. The authors use a pooled CRISPR/Cas9-based synergistic activation mediator screen to identify lncRNAs capable of regulating FOXO-mediated responses in cells. They identify a single lncRNA that binds to AKT and enhances AKT responses under basal conditions and upon growth factor stimulation. Further, the authors identify the DHX9 protein in the AKT/lncRNA complex, suggesting it as a novel regulator of AKT signaling. Overall, the paper makes some very interesting and novel observations regarding AKT signaling. This paper deserves publication in some form as it is also the first to describe a CRISPR-based activation screen using a lncRNA library. Furthermore, there are not many publications to use CRISPR genome editing for lncRNAs. However, there are serious concerns about the description of methodology that makes it very difficult to properly evaluate the quality of data. In particular, the methods are not described in sufficient detail. In addition, some aspects of the proposed model are not fully supported by the data.

We thank the reviewer for his/her positive comments.

Specific comments:

1. The Materials and Methods section lacks sufficient detail about the experiments. Most often, the authors refer to "according to manufacturer's instructions" or to the supplementary materials. But then again, there is not enough detail in the supplementary material section and again, referring mostly to manufacturer's protocols. Examples that are omitted include: which vectors were used, which cells (e.g. in the screen), the methods for identification of screen hits are not described properly (RNAseq?), mass spectrometry is not described, antibodies are not identified (e.g. catalogue or clone number is missing), CRISPR knockout generation is not described, the buffers used for protein isolation are missing, lentiviral transduction is not described. It is often not even clear which cells have been used.

Experimental details were added to the text or supplementary materials as follows.

There are three vectors used for the library screen, lenti sgRNA (MS2)-zeo backbone vector, dCas9-VP64 and pMS2-p65-HSF1 were obtained from Addgene. SAM gRNAs were cloned into lenti sgRNA (MS2)-zeo backbone vector. AK023948 and DHX9-myc tag were cloned into pCDH-MSCV-EF1-GFP-T2A-Pu (SBI). Vector for myc tagged p85 was pCDH-CMV-EF1-Pu. Vector for GST pulldown assays was pGEX-2T. Dual gRNA vector for AK023948 knockout and donor vector were described in NAR, 2015 Feb 18;43(3):e17.

Cell line used for library screening was MCF-7; other cell lines were indicated in figures or figure legend.

LncRNA profiling: qRT-PCR arrays.

Mass spectrometry analysis was provided by Applied Biomics. What we did was to cut out the unique band after silver staining and then sent to the company.

Antibody catalog # and clone number were included now.

Construction of AK023948 dual gRNA vector and donor vector was described in "Plasmid construction" section and more details were added to "AK023948 knockout by CRISPR/Cas9" section.

Lysis buffer for protein extraction and lentiviral transduction were provided in Supplementary materials.

2. Similarly, the Figure legends are very minimalistic and not of appropriate standard. This is a problem for all Figures throughout.

We added more information to each of the figure legends.

3. Figure 1A doesn't show much of "design". As it stands, the Figure does not show anything informative.

As suggested, we removed Figure 1A.

4. Figure 2D: it is not described what the top and bottom panels are.

We added "tumor tissue" to Figure 2D.

5. Figure 3B,C: is C the quantification of B?

They are different. Figure 3B is verification of the interaction between AK023948 and DHX9 by RNA precipitation, followed by Western blot using DHX9 antibody. Figure 3C is a result from immunoprecipitation, followed by qRT-PCR.

6. Legend for Figure 6 is not correct. (E) may be (D) and (F) is not described or wrongly labelled? What is meant by Figure 6(C) "bottom"? This is very confusing.

Sorry, this was our mislabeling. Figure D is tumor growth curves for vector control and AK0 KO#13 in nude mice (revised Fig. 4E). Figure E (revised Fig. 4F) is in situ hybridization for AK023948 in clinical tumor specimens. Figure C bottom indicated the time when in vitro cell growth was determined. We corrected them in the revised legend.

7. There is no validation of the CRISPR Knockouts. RT-PCR is mentioned in Figure S3. It would be good to know where the primers bind to, maybe shown in a scheme. The problem with CRISPR-mediated knockout of lncRNA is that validation has to be done at the RNA level. The authors are assuming that the dual gRNA design will result in a complete deletion of the entire transcript. However, this is not necessarily the case. One needs to evaluate whether both guides were efficiently processed. Also, whether both alleles (or multiple alleles, depending on the cell type - it would help to know which cells were used) are modified. It is even possible that there are only local insertion/deletions rather than loss of the entire locus. All this information will be useful for other researchers who plan to generate lncRNA

knockouts. For this, each allele for each clone needs to be sequenced at the nucleotide level surrounding the gRNA target sites.

We added more information to Fig.S3, including primers. We sequenced fragments involving deletions for these clones and detailed nucleotide sequencing information was shown in revised Fig. S4D.

8. The model presented in Figure 5F is not supported by the data. If the authors want to claim that AK023948 is required for binding of p85b to DHX9, this would need to be done with purified components in vitro. But then, Figure 5F is not cited in the text nor discussed.

Co-IP assay suggests that AK023948 is required for binding of p85 to DHX9 (Fig 4 C&D). Now we also performed GST pulldown assay which provides additional supporting evidence (revised Fig.4E) and PLA (revised Fig. 4F). We moved Figure 5F to supplementary figures as Fig. S17 and provided explanation in Discussion section in the revised manuscript.

9. The claim that p85alpha does not bind to the lncRNA is not fully supported. For instance, in Figure S6 the statement "No p85alpha was visible for pan-p85 antibody" is not backed by any data.

This is based on Figure S6A (revised Fig. S8A) which revealed that p85alpha was not detectable with p85alpha antibody in MCF-7 cells and Fig.4A showed that AK023948 KO suppressed the p85beta level, but had little effect on p85alpha. In Figure S6B (revised Fig. S8B) there was only a single band detected by pan-p85 antibody which matched with p85beta band, whereas no p85alpha band was detected with p85alpha antibody.

Minor points:
1. page 5: "cell singling" should be corrected.

Changed as suggested.

2. page 8, top: "... together they regulates..." should be "regulate".

Changed as suggested.

3. Page 8, top: "detectible" should be "detectable".

Changed as suggested.

4. page 10: "In consistent..." should be "In consistence...".

Changed as suggested.

Reviewer #2 (Remarks to the Author): Expert in lncRNAs

The present manuscript details a screen in which a number of lncRNAs were tested for the ability to positively regulate AKT. The top hit from the screen, AK023948 (AK0) lncRNA, was selected for further study. Through a combination of loss and gain of function study and physical association studies, the authors show that AK0 interacts with the RNA helicase DHX9, and that the stability of the oncogenic isoform of the regulatory subunit of PI3K, p85b, is compromised by the loss of function of either the lncRNA or the helicase. This, in turn, affected the activation of AKT through the PI3K axis. The presented data are interesting and novel, and the experimental approach taken are mostly appropriate. Further, the quality of the presented data is high and they are presented in a clear manner, except in Fig. 6 when the text and the figures do not match (e.g. Fig. 6C is not a tumor array). However, a number of issues have to be addressed to ensure the reproducibility of the data. Further, while the main conclusion of the manuscript, the involvement of AK0 and DHX9 in stability of p85, is well-supported, the interaction network between these three factors is not fleshed out. These issues are detailed below.

<u>We thank the reviewer for his/her positive comments.</u>

1) A major issue is the absence of sufficient controls in loss of function studies to ensure that the observed impacts don't stem from off-target effects in the case of siRNA approaches or clonal effects in the case of CRISPR-mediated KO approach. In all cases, more than one siRNA/KO cell line must be used, or, a rescue experiment must be shown. A number of important experiments in this study unfortunately lack these controls, which should be included.

<u>This reviewer's major concern is "off-target effect", which may occur in the case of siRNA or KO. As a matter of fact, we took several approaches to address this concern. First, we ectopically expressed AK023948 or knocked down AK023948 by RNAi. We also tested a second AK023948 siRNA and found a similar result (Fig. S3C). Second, we chose three KO clones to determine AK0 KO-mediated suppression of AKT activity (Fig. 2B). Third, we performed "rescue" experiments in two of three KO clones. Re-expression of AK023948 is able to rescue KO (Fig. 2C). If the observed phenotype, i.e., AK0 KO causes suppression of AKT activity, is due to off-target, this phenotype would not be rescued by re-expression of AK0. Together, all of these results suggest that off target is unlikely to play a role in this case.</u>

2) In the rescue/gain of function experiments when AK0 lncRNA or a protein-coding RNA is ectopically expressed, the level of overexpression compared to endogenous levels should be determined.

<u>These results were presented in Fig. S5A and Fig. 4G.</u>

3) Similarly, in all knock down studies, the extent of knock down should be determined.

<u>These results were presented in Figs. S3A&B and S11A.</u>

4) The physiological importance of the interaction between AK0 and DHX9 is an important unaddressed issue. Do the knock down or overexpression of DHX9 affect the stability or subcellular localization of AK0?

We showed that DHX9 knockdown had little effect on AK023948 level (Fig. S11A, bottom) so we did not test the stability of AK023948. In addition, DHX9 knockdown had little effect on the subcellular localization of AK023948 (Fig. S11B).

5) Similarly, to understand the mechanism of the observed regulatory impact, the mode of interaction of DHX9 and AK0 with p85 should be better fleshed out. Do they both directly interact with p85, or one alters the conformation of the other so it can interact with p85? An informative experiment in this regard could be mapping the region in AK0 that is needed for interaction with DHX9 and p85. Do these interactions map to different regions of the RNA (suggesting that the lncRNA acts as a scaffold) or the same region (which could mean that AK0 indirectly interacts with p85 through DHX9)?

We made deletions for AK023948. RNA precipitation assay showed that both DHX9 and p85 can interact with the second fragment (AK0-2). The result was shown in Fig. S9A. Since this fragment is still relatively large, we cannot tell at this point whether they interact with AK023948 at the same or different regions.

6) Please correct the text/figure discord in Fig. 6.

We made corrections in revised Fig. 6 legend.

Minor points:

1) p3, line 38. lncRNAs are not usually capped and poly-A-ed. A large fraction of lncRNAs are not polyadenylated.

Changed as suggested.

2) In Fig. 2C, does the rescue experiment restore the level of pAKT to the level in naive cells?

Yes, see Fig. S5A which showed pAKT level in gRNA control cells as compared to rescued cells. In addition, we found that the pAKT level is about same in both naïve cells and gRNA control cells.

3) Page 8, first paragraph. Data in Fig. S6A does not provide any information about the relative abundance of the two isoforms. The difference in the signal intensity cannot be compared for different antibodies or even the same antibody on the same blot, as the affinity of the antibody for the two targets may be different. The same is true for the other estimations of abundance made based on signal intensity of western blots.

The reviewer is right. All we can say is that we detect a low level of this protein with this specific antibody. Thus, we changed our wording in the revised manuscript.

Reviewer #3 (Remarks to the Author): Expert in AKT signalling in cancer

AKT is a protein serine/threonine kinase that plays a central role in the regulation of various cellular processes, particularly survival and metabolism. It is activated downstream of PI3K, due to phosphorylation of its Thr308 by PDK1 and Ser473 by mTORC2. Importantly, AKT is often dysregulated in cancer, however, the underlying mechanism is still not fully understood. Here, the authors show that the LncRNA, AK023948, acts as a novel positive regulator of the PI3K/AKT pathway. Moreover, they provide evidence that AK023948 exert its function by interacting the ATP dependent RNA helicase A (RHA/DHX9) and the regulatory subunit of PI3K, p85beta. These interactions stabilize PI3K and therefore can sustain high basal activity of AKT.
Generally, the manuscript is well written, presenting important and interesting new data. However, some of the conclusions drawn regarding the molecular mechanism of action are not fully substantiated and some other points need to be addressed in order to make this study suitable for publication. These are as follows:

We thank the reviewer for his/her positive comments.

1. The authors hypothesize that LncRNA AK023948 exert its function by interacting with DHX9 and p85, which stabilizes the latter. However, it is not clear to me that the small differences in p85 expression are sufficient in order to induce AKT activation. Moreover, it is possible that AK023948 and DHX9 interact with additional upstream components to cause the effects. Therefore, It is suggested to modulate p85beta expression by SiRNA or overexpression in order to verify that the effect is indeed mediated by the stabilization of p85.

As suggested, we performed p85 beta siRNA knock down assay. As expected, suppression of p85 beta by RNAi significantly reduced the pAKT level (Fig. S10B).

2. Moreover, more experiments are required in order to verify the existence/formation of the AK023948/DHX9/p85 heterotrimer. For example, sizing column that will show whether this complex is formed by itself, or whether it interact with additional proteins. In addition, the interaction between the proteins is based on CoIP experiments only. The authors should use additional methods to confirm their point, such as proximity ligation assay (PLA) or FRET.

Yes, it would be good to know all the components of this complex, but this could be very complicated. We made a few attempts for sizing column but was not successful probably because we do not have this kind of expertise. We would like to pursue in the future. Regarding the interaction between DHX9 and p85, we did show their interaction by GST pulldown assay (Fig. 4E). Please also see our response to comment # 3 from reviewer 4.

As suggested, we also performed PLA using Sigma's Duolink in situ fluorescence kit. We showed that AK0 siRNA can reduce the interaction between DHX9 and p85. The results were presented in revised Fig.4F and Fig.S9B.

3. The authors showed that PTEN is not involved in the suppression of pAKT. It would be important to check whether the effect is mediated by PP2A and PHLPP that regulate AKT phosphorylation directly.

We used the PP2A inhibitor okadaic acid for this experiment and found that okadaic acid can induce AKT in both gRNA control and AK0 KO cells, but the level of AKT activation is lower in KO than control cells. The result was shown in Fig. S12B.

4. It is clear from the results that AK023948 affects AKT phosphorylation. However, it was not mentioned what residue was followed (T308 or S473), and whether there was a significant change in the activity of AKT. If the effect was just on T473, it is possible that the actual activity of AKT was not change, and may lead to distinct conclusion regarding the mechanism of AK023948 action.

The phosphorylation residue is now shown in the corresponding pAKT blots. Moreover, we also show that AK0 KO cause a reduction of phosphorylated PDK1 (Fig. S5B) which is responsible for phosphorylation of T308 at AKT. These results suggest that AK0-mediated AKT activity is through PI3K.

5. Many of the changes presented in the blots are very small, and it is not clear how significant they are. The authors should quantify their results, and include statistical analysis and significance to all figures.

We provided quantitative data for western blots in additional supplementary materials.

6. An interesting question that hasn't been addressed is whether the knock out of AK023948 induces cell apoptosis that is usually seen when AKT activity is reduced. This would provide a strong indication on the significance and robustness of the AK023948 effect.

As suggested, we performed TUNEL assay. Indeed, AK0 KO promotes H_2O_2-induced apoptosis. The result was shown in revised Fig. 6D and Fig. S14.

7. The starvation used in Fig. 5 (2 hours) is very problematic, as the change of medium/serum by itself is known to induce rapid ERK and AKT activation, and a later induction of feedback inhibition. It is very likely that the effects here were measured when the negative feedback was maximal. Therefore, the authors should use at least 12 hours starvation (or no starvation at all). In addition, the authors should include more time points (5-90 min) to the EGF and Ins stimulation, as it is possible that some effects, especially on ERK will be seen at different time points.

As suggested, we performed additional experiments with 12 h starvation of cells and then followed the time course from 5-90 min after addition of insulin. There was an obvious induction of AKT in gRNA control by insulin, but such induction was not so obvious in AK0 KO cells. On the other hand, the difference of ERK induction between gRNA control and AK0 was not obvious. The result was shown in Fig. S12A.

8. In Figure S6C, please blot for p-AKT as well.

As suggested, we examined both pAKT and total AKT under the same condition. We found that pAKT level was increased at later time points (3~6 h) in control siRNA cells and the upregulation of pAKT was also seen at 6 h in DHX9-siRNA cells (revised Fig. S10A). Upregulation of pAKT after cycloheximide treatment is consistent with a report that cycloheximide itself can also induce AKT activity (J Biol Chem. 2013 Aug 16;288(33):23875-83).

9. Some of the "signaling terminology" is problematic, and more details should be added to the section on AKT in the introduction.

We tried our best to describe PI3K/AKT signaling and changes were made in revised introduction section. Hope that it is better now.

10. Page 9 line 186- the reference was probably supposed to be towards figure 6E and not 6C as indicated.

Changed as suggested.

11. Page 10 line 194- the reference was probably supposed to be towards figure 6D and not 6E as indicated.

Changed as suggested.

12. I am not an expert in the field of LncRNA, but I found it surprising that the authors did not describe the genomic location of AK023948. Looking at it, I found that AK023948 is all within an intron of two other genes, which is probably problematic for expression, as well as SiRNA or CRISPR targeting. These points should be properly discussed.

Yes, the reviewer right that AK023948 is imbedded in thyroglobulin (TG) and Src-like-adaptor (SLA). TG consists of 48 exons, and AK023948 is located in the intron (~65kb) between exon 41 and 42 in the same direction and it is also in the intron (9,184 bp) of SLA between exon 3 and 4 in an opposite direction. To determine whether AK0 KO affects these two genes, we designed primers covering TG exon 41 and 42, and SLA exon 3 and exon 4. qRT-PCR analysis revealed that TG expression is undetectable in both gRNA control and KO cells; and SLA expression is detectable, but AK0 KO had no effect on SLA (Fig. S5C), suggesting that AK0 has own its promoter.

Reviewer #4 (Remarks to the Author): Expert in breast cancer

Suggested decision: major revise
Comments:
The manuscript entitled "lncRNA AK023948 is a positive regulator of AKT" identified lncRNA AK023948 as a positive regulator of AKT pathway by using synergistic mediation activator (SAM) and AKT reporter system. Mechanismly, the authors showed AK023948 could bind DHX9 and mediated interaction between DHX9 and p85, which increase the stability of p85. This work provide a linkage between lncRNA and AKT pathway, which will deepen our understanding of lncRNA and AKT pathway. Moreover, the screening methods described in this manuscript is very useful for investigation of roles of lncRNA in other important pathways. But there are some critical issues that should be addressed by the authors:

We thank the reviewer for his/her positive comments.

1. Inverstigation of some basic information of AK023948 shows AK023948 is an intronic lncRNA that located in the intron of TG(thyroglobulin) gene, interestingly it is also in the intron of another gene, Src-like-adaptor (SLA). AK023948 has the same direction with TG and opposite direction of SLA. It is reported that src-like adaptor Protein (SLAP) binds to the receptor tyrosine kinase Flt3 and has impact on FL-induced PI3K-Akt pathway. The authors used CRISPR/Cas9-based method to overexpress or knock out this lncRNA, it is very likely that these genome-editing methods may also influence the host gene, e.g., transcription and/or splicing. The authors should demonstrate CRISPR/Cas9 and siRNAs have no impact on host genes to rule out the possibility that activation of AKT is caused by host gene rather than AK023948. Furthermore, it is suggested the authors should provide subcellular localization and the full-length imformation (using 5'RACE and 3'RACE) of AK023948.

Please see our response to comment #12 from Reviewer 3 for the first part.

Regarding subcellular localization of AK023948, we found that AK023948 is present in both cytoplasm and nucleus as determined by FISH (Fig. S11B).

Regarding the full-length information of AK023948, we confirmed the AK023984 transcript by 5'RACE and 3'RACE, in consistence with GenBank: AK023948.1.

2. Since AK023948 activates AKT is the most important finding in this work, the authors should give more evidence to show AKT pathway is truly influenced by AK023948, e.g., the AKT reporter activity and downstream gene expression. Moreover, the authors did not mention which phosphorylation site of AKT was detected.

Regarding AKT reporter activity, Fig. S2C is an AKT reporter assay where activation of AKT by AKO SAM gRNAs confers resistance to puromycin.

In addition, we examined apoptosis, a downstream effect of AKT. AK0 KO promotes H_2O_2-induced apoptosis. The results were shown in revised Fig. 6D and Fig. S14.

Regarding AKT phosphorylation sites, we found that AK023948 activates AKT at both T308 and S473 sites. Please also see our response to comment #4 from Reviewer 3.

3. The authors suggested AK023948 can as a scaffold of DHX9 and p85, but p85 can bind AK023948 was only proved by RIP-qPCR, the authors should use the same RNA precipitate used in Figure 3A and 3B to detect p85 by western bolt. Furthermore, DHX9 co-IP to show DHX9 can interact with p85, since this interaction is very important for AKT activation, the authorsed should use in vitro study like GST-pull down experiments (with or without AK023948) to further demonstrate DHX9 and p85 can interact in vitro and AK023948 is essential for the interaction.

Please see Fig. S9A for RNA precipitation results. We detected p85 protein by both full length AK0 and AK0-2.

As suggested, we performed GST pulldown assays. Consistent with co-IP results, AK023948 is required for the interaction between DHX9 and p85. The result was presented in revised Fig. 4E

4. In Figure 1, Figure 2, Figure 3 and Figure 5C, total ATK potein is used as control, the authors should use housekeeping gene for loading control to show AK023948 have no impact on total AKT.

We have added GAPDH to these figures.

Reviewers' comments:

Reviewer #1 (Remarks to the Author):

Thank you for the clarifications. I am happy with the changes, just have two minor points: 1. I still don't see how Figure S8B shows that p85b is the predominant form. I think Figure S8A is sufficient to show and the statement should be removed. 2. The images in Figure S11B are very poor quality. It would be good to increase quality.

Reviewer #2 (Remarks to the Author):

While the authors have prepared a significantly improved revised manuscript, there are still some major concerns left regarding the scientific validity of the data shown, plus minor needed changes to improve the clarity of the manuscript, as detailed below.

1) The issue of off-target effect of knock down studies and clone-specific effect for knock out studies is still not sufficiently addressed. While one or two experiments have been performed with sufficient controls, the vast majority of the studies shown are performed with only a single KO clone (#13) or with a single siRNA without any rescue experiments, these include Figures 2F, 4A, 4D, 4F, 5B, 5C, 5D, 5E, 5F, 6D and 6E, and many figures in the supplementary data provided (e.g. S12A, B, S15, etc).

The authors need to be aware that the rescue of just one aspect of the phenotype by overexpression or the presence of just one aspect of the phenotype in multiple KO clones are NOT a guarantee that the rest of the impacts shown by the authors will be similarly reproducible in different KO clones/knock down studies. So the data shown in Fig. 2B, 2C and S3C don't prove that the rest of their observations are similarly a general property of loss of the lncRNA/proteins knocked out/down. This is a very major concern, since it's entirely possible (in a worst case scenario) that the majority of the presented data is specific to KO clone 13 and not reproducible in other KO clones. Such clone specific effects sadly have been observed in other studies and as the authors know, low levels of reproducibility in published scientific work is becoming a major issue and frequently stems from a lack of sufficient controls. Thus, to clarify that their observations don't stem from a clone-specific phenotype, several key experiments (in addition to those in Fig. 2B,C and S3C) need to be performed in more than one KO clone or with multiple siRNAs in the case of knock down studies.

3) In response to my 3rd comment, the authors have added Fig. S11A, however, the figure legend does not clarify what is shown in this panel. Two different experiments done on different dates? Two different siRNAs? Two different cell lines/clones?

4) Similarly, what is shown in Fig. 4E? Are these two repeats? Two different KO clones?

Reviewer #3 (Remarks to the Author):

The authors addressed all my concerns. The manuscript is much improved now, and I have no further comments.

Reviewer #4 (Remarks to the Author):

In this revised manuscript, the authors had well addressed all the critiques raised by the reviewers by providing several new data panels and literature review. The manuscript is now suitable for publication in Nature Communication.

Reviewers' comments:

Reviewer #1 (Remarks to the Author):

Thank you for the clarifications. I am happy with the changes, just have two minor points:

1. I still don't see how Figure S8B shows that p85b is the predominant form. I think Figure S8A is sufficient to show and the statement should be removed.

As suggested, we removed the statement.

2. The images in Figure S11B are very poor quality. It would be good to increase quality.

We did one more time, but this was the best we could get. Nevertheless, the relative distribution of AK023948 is visible.

Reviewer #2 (Remarks to the Author):

While the authors have prepared a significantly improved revised manuscript, there are still some major concerns left regarding the scientific validity of the data shown, plus minor needed changes to improve the clarity of the manuscript, as detailed below.

1) The issue of off-target effect of knock down studies and clone-specific effect for knock out studies is still not sufficiently addressed. While one or two experiments have been performed with sufficient controls, the vast majority of the studies shown are performed with only a single KO clone (#13) or with a single siRNA without any rescue experiments, these include Figures 2F, 4A, 4D, 4F, 5B, 5C, 5D, 5E, 5F, 6D and 6E, and many figures in the supplementary data provided (e.g. S12A, B, S15, etc).

We understand the reviewer's concern that our major finding, i.e., AK023948 promotes AKT activity, might be an artifact or clone specific. RNAi and KO approaches are complementary in nature, and these experiments were performed in different cells. Furthermore, we characterized three KO clones for their effect on AKT and obtained consistent results. More importantly, we performed a key experiment, i.e., rescue experiment, in two clones. Regarding siRNA without any rescue experiments, unlike KO with which we can perform rescue experiments, but it is extremely difficult to perform rescue for siRNA because the introduced cDNA (rescue) will also be subject to targeting by the corresponding siRNA.

The authors need to be aware that the rescue of just one aspect of the phenotype by overexpression or the presence of just one aspect of the phenotype in multiple KO clones are NOT a guarantee that the rest of the impacts shown by the authors will be similarly reproducible in different KO clones/knock down studies. So the data shown in Fig. 2B, 2C and S3C don't prove that the rest of their observations are similarly a general property of loss of the lncRNA/proteins knocked out/down. This is a very major concern, since it's entirely possible (in

a worst case scenario) that the majority of the presented data is specific to KO clone 13 and not reproducible in other KO clones. Such clone specific effects sadly have been observed in other studies and as the authors know, low levels of reproducibility in published scientific work is becoming a major issue and frequently stems from a lack of sufficient controls. Thus, to clarify that their observations don't stem from a clone-specific
phenotype, several key experiments (in addition to those in Fig. 2B,C and S3C) need to be performed in more than one KO clone or with multiple siRNAs in the case of knock down studies.

We performed these additional experiments and confirmed that the same phenotype seen in clone #13 was also seen in clone #28 or AK0 siRNA2

Suppression of p85β in KO#28 (see modified Fig. S9A).

Inhibition of the EGF/insulin-induced AKT activation by AK0 KO in clone #28 (see modified Fig. S12A).

AK0 siRNA2 suppresses pAKT in BT549 cells (see modified Fig. S12D).

AK0 KO promotes H_20_2-induced apoptosis in clone #28 (see modified Fig. 6D and Fig. S14, right).

3) In response to my 3rd comment, the authors have added Fig. S11A, however, the figure legend does not clarify what is shown in this panel. Two different experiments done on different dates? Two different siRNAs? Two different cell lines/clones?

They were done on different days in MCF-7 cells with the same siRNAs (pooled 3~4 siRNAs).

4) Similarly, what is shown in Fig. 4E? Are these two repeats? Two different KO clones?

They are two clones (#13 and #28).

Reviewer #3 (Remarks to the Author):

The authors addressed all my concerns. The manuscript is much improved now, and I have no further comments.

Reviewer #4 (Remarks to the Author):

In this revised manuscript, the authors had well addressed all the critiques raised by the reviewers by providing several new data panels and literature review. The manuscript is now suitable for publication in Nature Communication.

REVIEWERS' COMMENTS:

Reviewer #1 (Remarks to the Author):

I am happy with the changes and recommend publication of the article.

Reviewer #2 (Remarks to the Author):

The added data address the major concerns this reviewer had with the last version of the manuscript. However, there are some minor changes in the text that should be made to ensure clarity.

1) The legend to Fig. S10 needs clarification: section B (not D, please correct the typo) needs to include information provided by the authors in response to reviewer letter, that is, it should be clarified how the two side-by-side repeats differ from each other. If they are done on different dates/different batches of cell/etc, please indicate this in the legend.

2) Same as above, for Fig. S11A.

3) Either it should be indicated that except when stated, a designation of KO in figure labels/text/figure legends means KO clone 13, or all the KO labels should clarify which clone is being studied. For example, Fig. S12B.

Responses to reviewer 2: minor points

1) The legend to Fig. S10 needs clarification: section B (not D, please correct the typo) needs to include information provided by the authors in response to reviewer letter, that is, it should be clarified how the two side-by-side repeats differ from each other. If they are done on different dates/different batches of cell/etc, please indicate this in the legend.

Changed as suggested.

2) Same as above, for Fig. S11A.

Changed as suggested.

3) Either it should be indicated that except when stated, a designation of KO in figure labels/text/figure legends means KO clone 13, or all the KO labels should clarify which clone is being studied. For example, Fig. S12B.

We labeled all KO clones in figures and figure legends.
